# Ketogenic Metabolic Therapy, Without Chemo or Radiation, for the Long-Term Management of *IDH1*-Mutant Glioblastoma: An 80-Month Follow-Up Case Report

**DOI:** 10.3389/fnut.2021.682243

**Published:** 2021-05-31

**Authors:** Thomas N. Seyfried, Aditya G. Shivane, Miriam Kalamian, Joseph C. Maroon, Purna Mukherjee, Giulio Zuccoli

**Affiliations:** ^1^Biology Department, Boston College, Chestnut Hill, MA, United States; ^2^Department of Cellular and Anatomical Pathology, University Hospital Plymouth National Health Service (NHS) Trust, Plymouth, United Kingdom; ^3^Dietary Therapies LLC, Hamilton, MT, United States; ^4^Department of Neurosurgery, Medical Center, University of Pittsburgh, Pittsburgh, PA, United States; ^5^Department of Radiology, St. Christopher Hospital for Children, Drexel University School of Medicine, Philadelphia, PA, United States

**Keywords:** standard of care, glycolysis, glutaminolysis, fasting, mitochondrial substrate level phosphorylation (mSLP), 2-hydroxyglutarate, carnivore diet

## Abstract

**Background:** Successful treatment of glioblastoma (GBM) remains futile despite decades of intense research. GBM is similar to most other malignant cancers in requiring glucose and glutamine for growth, regardless of histological or genetic heterogeneity. Ketogenic metabolic therapy (KMT) is a non-toxic nutritional intervention for cancer management. We report the case of a 32-year-old man who presented in 2014 with seizures and a right frontal lobe tumor on MRI. The tumor cells were immunoreactive with antibodies to the *IDH1* (R132H) mutation, P53 (patchy), MIB-1 index (4–6%), and absent ATRX protein expression. DNA analysis showed no evidence of methylation of the MGMT gene promoter. The presence of prominent microvascular proliferation and areas of necrosis were consistent with an *IDH*-mutant glioblastoma (WHO Grade 4).

**Methods:** The patient refused standard of care (SOC) and steroid medication after initial diagnosis, but was knowledgeable and self-motivated enough to consume a low-carbohydrate ketogenic diet consisting mostly of saturated fats, minimal vegetables, and a variety of meats. The patient used the glucose ketone index calculator to maintain his Glucose Ketone Index (GKI) near 2.0 without body weight loss.

**Results:** The tumor continued to grow slowly without expected vasogenic edema until 2017, when the patient opted for surgical debulking. The enhancing area, centered in the inferior frontal gyrus, was surgically excised. The pathology specimen confirmed *IDH1-*mutant GBM. Following surgery, the patient continued with a self-administered ketogenic diet to maintain low GKI values, indicative of therapeutic ketosis. At the time of this report (May 2021), the patient remains alive with a good quality of life, except for occasional seizures. MRI continues to show slow interval progression of the tumor.

**Conclusion:** This is the first report of confirmed *IDH1*-mutant GBM treated with KMT and surgical debulking without chemo- or radiotherapy. The long-term survival of this patient, now at 80 months, could be due in part to a therapeutic metabolic synergy between KMT and the *IDH1* mutation that simultaneously target the glycolysis and glutaminolysis pathways that are essential for GBM growth. Further studies are needed to determine if this non-toxic therapeutic strategy could be effective in providing long-term management for other GBM patients with or without *IDH* mutations.

## Introduction

Glioblastoma (GBM) has among the highest mortality rates for primary brain tumors and remains poorly manageable. Despite the hype surrounding recent therapies (1–5), median life expectancy following diagnosis remains poor for most GBM patients (6–9). Survival is slightly better, however, for younger GBM patients compared to older GBM patients and for patients with GBM tumors that express *IDH1* mutations (10–14). Most GBM patients receive the current standard of care (SOC) involving surgical debulking, radiotherapy, and temozolomide (TMZ) chemotherapy (15, 16). Many GBM patients can also receive corticosteroid medication (dexamethasone) and bevacizumab for managing edema and angiogenesis, respectively. Use of steroids is now under serious reevaluation, as steroids can elevate blood glucose, which is associated with more rapid tumor growth and shortened overall survival (17, 18). The current SOC for GBM has only marginally improved overall survival compared to “best supportive care,” which is ambiguous at best (19, 20). Equally distressing to management failure is evidence that the incidence of GBM is increasing in the United Kingdom (21).

We recently reviewed studies describing the adverse effects that can be associated with the current SOC for GBM management (17). GBM, like most malignant cancers, is driven by glucose and glutamine fermentation through the glycolysis and glutaminolysis pathways, respectively (22–27). The dependency on glucose and glutamine fermentation arises from inefficient oxidative phosphorylation (OxPhos) that is linked to abnormalities in the number, structure, and function of mitochondria in GBM tissue (17, 26–33). Surgical debulking followed by radiotherapy inadvertently increases the availability of glucose and glutamine in the tumor microenvironment (17, 34, 35). TMZ chemotherapy can further damage mitochondria while, at the same time, increasing systemic inflammation and tumor driver mutations (36, 37). Bevacizumab is even more likely than TMZ to cause mitochondrial dysfunction in human brain tumors, and is remarkable in its ability to facilitate distal tumor cell invasion through the neural parenchyma and the perivascular network (38–40). Use of dexamethasone can further accelerate GBM growth by increasing blood glucose levels and glutamine metabolism (25, 41–44). In light of this information, the poor progression free and overall survival experienced by most GBM patients receiving the SOC should not be surprising.

Winter and colleagues coined the term “Ketogenic Metabolic Therapy (KMT)” to describe an anti-neoplastic nutritional strategy, using ketogenic or low-glycemic diets, for the management of malignant gliomas (45). KMT is gaining recognition as a complementary therapeutic strategy for the management of a broad range of cancers in addition to malignant gliomas (17, 19, 45–60). Low carbohydrate, high fat ketogenic diets (KD) reduce the glucose needed to drive aerobic fermentation (Warburg effect) while also elevating ketone bodies, which cannot be effectively metabolized for energy in tumor cells due to defects in mitochondrial OxPhos (17, 26, 45, 56, 61–67). Moreover, ketone body metabolism enhances the ΔG′ATP hydrolysis in normal cells from −56kJ/mole to −59kJ/mole, thus providing normal cells with an energetic advantage over tumor cells (27, 68, 69). Calorie restriction and restricted KD are also anti-angiogenic, anti-inflammatory, anti-invasive, and can kill tumor cells directly through pro-apoptotic mechanisms (17, 62, 70–73). Evidence also shows that therapeutic ketosis can act synergistically with several drugs and procedures to enhance cancer management thus improving both progression free and overall survival (26, 74–76). Hence, KMT targets the multiple drivers of rapid GBM growth while enhancing the metabolic efficiency of normal brain cells (56).

## Case Report

**Figure 1:**
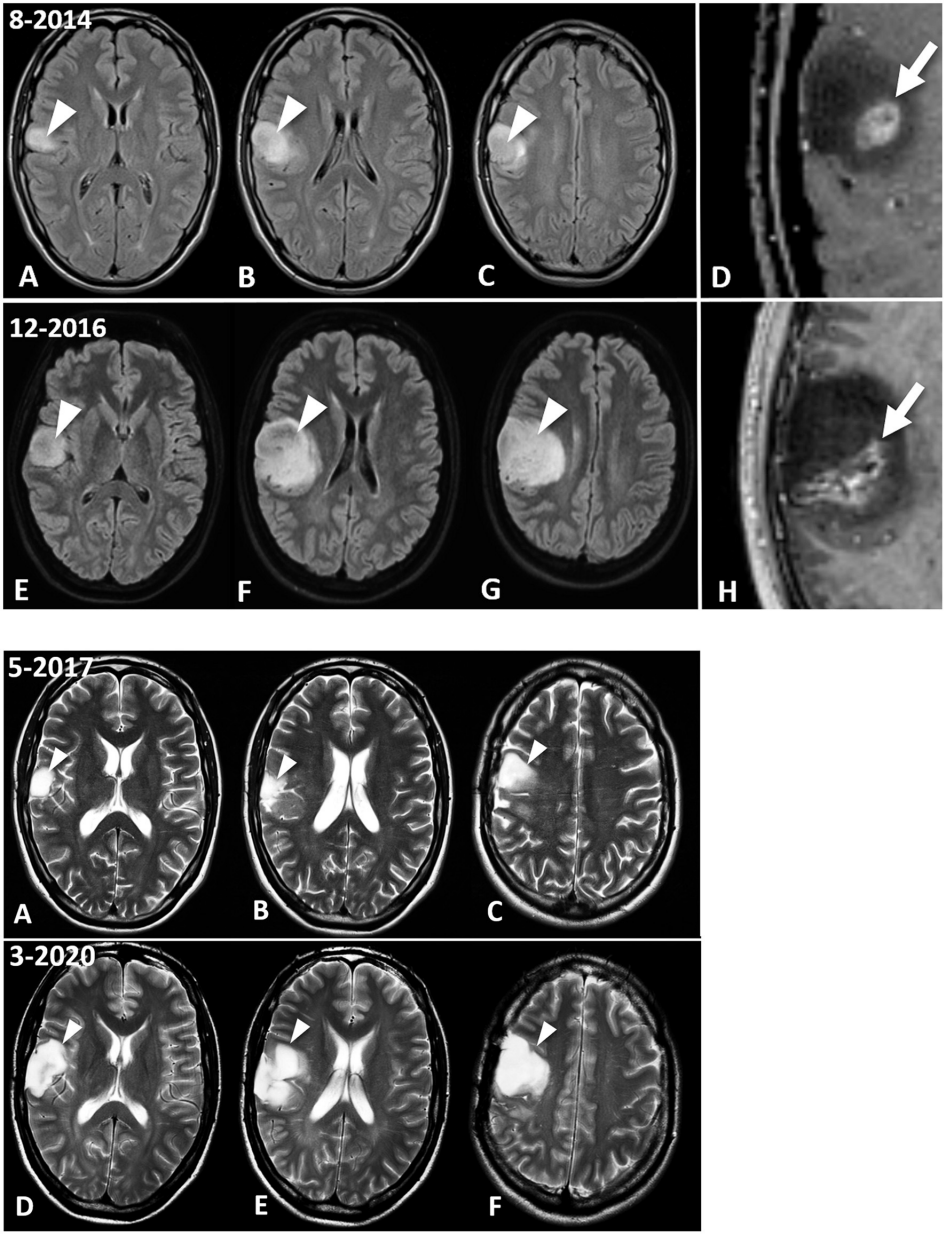
MRI images of the patient's brain tumour. **Panel 1**. Fluid Attenuated Inversion Recovery (FLAIR) images show the *IDH1*-mutant GBM at diagnosis in August 2014 (A–D) and ~8 months later, at the last follow-up MRI examination (December 2016) prior to surgical excision of the enhancing nodule (E–H). The lesion discovered on August 2018 is centered in the right inferior frontal lobe (A,B, arrowheads), and abuts the premotor cortex (C, arrowhead). There is an enhancing nodule within the lesion, as seen in the magnified post-contrast image (D) (arrow). This enhancing lesion measured 1.25 mL, which is calculated using the V = ABC/2 formula. Follow-up MRI (E–H) demonstrates interval progression of the non-enhancing tumor (arrowheads). Interval increase in size of the enhancing lesion, measuring 5.97 mL, was also observed (H, arrow). **Panel 2** shows the evolution of the lesion between the May 2017 surgical excision (A–C), and in the most recent MRI evaluation, dated March 2020 (D–F). T2-weighted images indicate the residual GBM (arrowheads). (B) shows the T2-hyperintense GBM filling the surgical cavity. The lesion in the premotor cortex is clearly seen in (C). Please note that the surgical debulking involved only the enhancing tumor, while the largest non-enhancing part of the GBM was not excised by the neurosurgeon. The most recent brain MRI (D–F) shows interval increase in size of the GBM (arrowheads), which remains circumscribed to the right frontal region without infiltrating the white matter tracts.

A 26-year-old male from South Devon presented on August 16, 2014 at University Hospitals Plymouth NHS Trust, PL6 8DH, UK, with two episodes of left-sided facial numbness and bilateral tonic-clonic seizures originating from the right temporal lobe. There was no history of malignancy or chronic disorders. The patient's blood pressure was within normal limits (110/70). Laboratory investigation revealed unremarkable blood chemistry, with liver and renal functions within normal limits. Fasting blood glucose and C-Reactive protein were within normal ranges. Prior to therapeutic intervention, the patient's weight, height, and body mass index (BMI) were 63 kg, 180 centimeters, and 19.4 kg/m^2^, respectively. The contrast enhanced brain MRI (August 22, 2014) showed an intra-axial lesion, centered in the right inferior frontal lobe. The lesion, which was mainly non-enhancing and solid, disclosed the presence of an eccentric contrast enhancing nodule (Figure 1, **Panel 1**, D).

**Figure 2:**
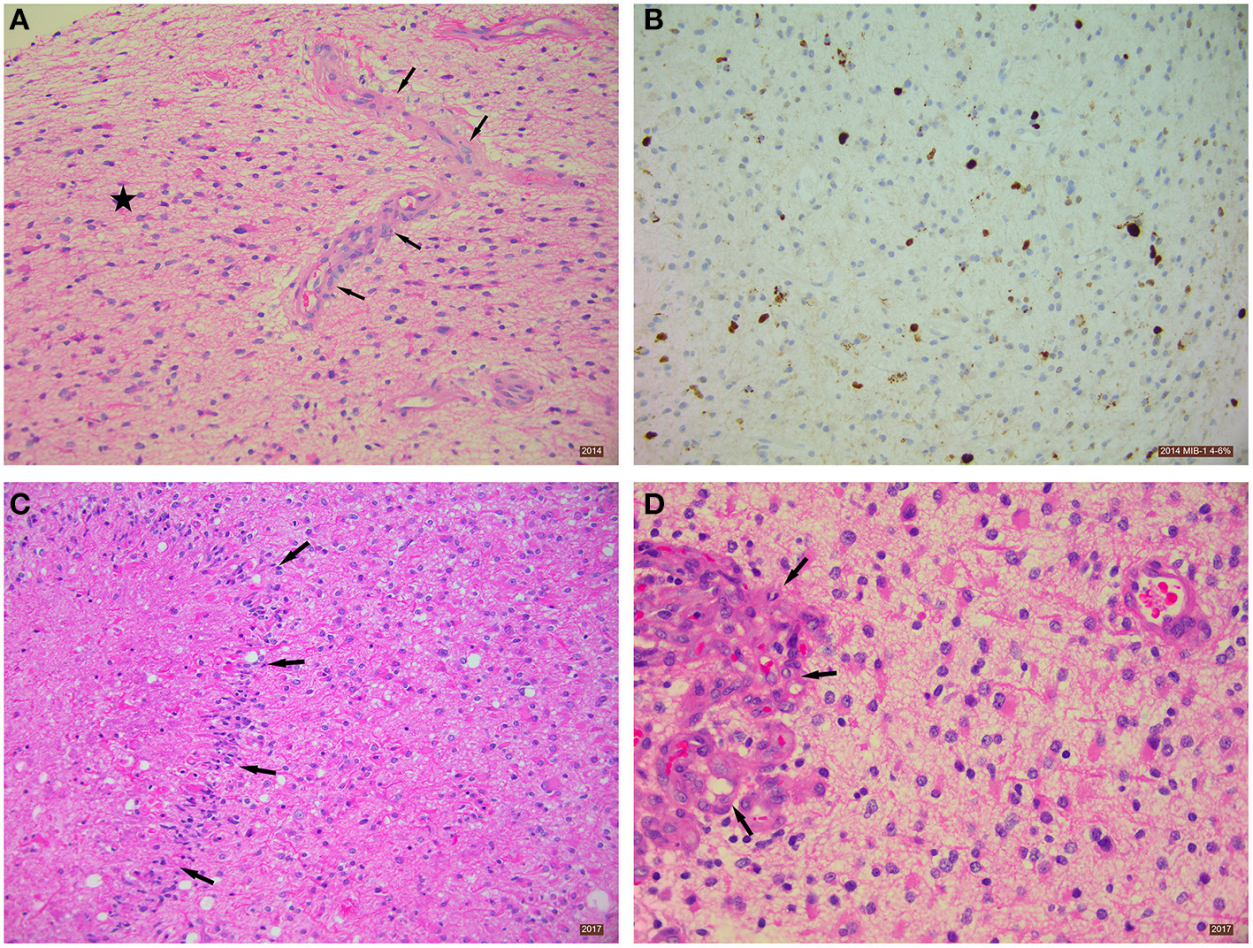
Histopathological analysis of excised brain tumor tissue. **(A)** Diffusely-infiltrating astrocytic tumor *(star)* with focal microvascular proliferation *(arrows)*. Tissue taken from 2014 biopsy, H&E stain 200x. **(B)** Immunohistochemistry using MIB-1 antibody showing a proliferation index of ~4–6%. Tissue taken from 2014 biopsy, Immunostain 200x. **(C)** Section showing necrosis and palisading of astrocytic tumor cells around the necrotic area *(arrows)*. Tissue taken from 2017 biopsy, H&E stain 200x. **(D)** Section showing glomeruloid vascular proliferation *(arrows)*. Tissue taken from 2017 biopsy, H&E stain 400x.

Enhanced MRI of the brain (August 22, 2014) showed a lobar T2 signal abnormality without restricted diffusion and with central ring enhancement centered on the opercula portion of the inferior frontal gyrus. Signal change also extended into the precentral gyrus (Figure 1, **Panel 1**, C). The preliminary impression was a transforming low-grade glioma. Histopathological analysis (September 16, 2014) of seven cream and white cores of brain biopsy tissue revealed a diffuse cortical infiltration by a paucicellular glial neoplasm composed of predominantly fibrillary and occasional gemistocytic astrocytes. The tumor cells were moderately pleomorphic with infrequent/rare mitosis. There was focal micro-calcification and focal microvascular proliferation (Figure 2A), but no necrosis was about present. The MIB-1 (Ki67) proliferation index was 4–6% (Figure 2B). The tumor cells were immune-reactive with antibodies to mutant *IDH1* (*R132H*) and to patchy P53 expression. DNA analysis showed no evidence of the MGMT gene promoter methylation. The presence of rare mitoses, focal microvascular proliferation and MIB-1 proliferation index was compatible with an *IDH*-mutant glioblastoma (WHO Grade 4).

Due to the patient's cultural beliefs regarding toxic therapies, he refused the recommended SOC. Instead, he opted for self-administering ketogenic metabolic therapy (KMT) that was initiated 2 weeks after the histopathological diagnosis of GBM. The patient was motivated to educate himself on proper implementation of the diet, replacing the recommended SOC with KMT despite pressure from his healthcare providers to use SOC. The energy composition of his daily diet consisted of fat (1,696 kcal), protein (264 kcal), and carbohydrates (48 kcal) with the addition of MCT oil (medium chain triglycerides). He was prescribed levetiracetam (750 mg, 2x/day) for seizure management, and MCT oil (3 tsp daily with food). He strictly followed the ketogenic diet guidelines found on Patricia Daly's website (https://patriciadaly.com/the-ketogenic-diet-for-cancer), and used the Precision Xtra glucose/ketone meter (Abbott Labs) and the glucose/ketone index calculator to monitor his blood glucose (mmol) and blood β-hydroxybutyrate (β-OHB) (mmol) values (77). It took the patient 2 weeks to enter the predicted zone of therapeutic ketosis, i.e., glucose/ketone index (GKI) values near 2.0 or below, as previously described (77). A second MRI of his tumor conducted on January 24, 2015 revealed no noticeable progression.

Serial MRIs preformed on April 14, 2015; July 17, 2015; November 16, 2015; February 20, 2016; July 9, 2016; and October 29, 2016 revealed evidence of interval slow contrast-enhanced tumor progression over that seen on the original 2014 MRI. MRI evidence of contrast-enhancing disease progression was more concerning on the follow-up MRI from December 15, 2016 (Figure 1, **Panel 1**, H). In response to these observed changes, the patient opted for an awake debulking craniotomy in April 2017. Excision was uneventful, resulting in gross complete removal of the mixed solid-necrotic contrast enhancing component of the GBM. However, the largest T2 hyperintense part of the GBM remained untouched (Figure 1, **Panel 2**, A–C). Histological analysis of the tumor tissue showed a diffusely invasive astrocytic tumor with infrequent/rare mitosis, prominent microvascular proliferation (Figure 2C) and areas of necrosis (Figure 2D). The tumor cells expressed mutant *IDH1* (R132H), P53 (patchy), and showed loss of nuclear ATRX expression. The overall histological features were in keeping with the diagnosis of *IDH1-mutant* glioblastoma (WHO Grade 4).

The patient continued with a strict ketogenic diet regimen following tumor debulking and maintained his GKI values at or near 2.0 and below. The following medications were taken for 1 week only after the craniotomy and included Epilim (200 mg), dexamethasone (2 mg), omeprazole (20 mg), and paracetamol (1 g) for post-surgical pain management. Tonic-clonic seizure activity, which increased after the craniotomy, gradually subsided over time. Various supplements were added to the diet that included vitamins, minerals, turmeric, resveratrol, omega-3, and boswellia serrata. No further tumor growth was seen on the MRI preformed on August 17, 2017. As the patient believed that his GBM was under control, he relaxed his adherence to low-carbohydrate foods. This resulted in modest body weight gain (89 kg) and elevated his GKI values to the 5–10 range indicative of increased blood glucose and reduced ketone levels.

**Figure 3:**
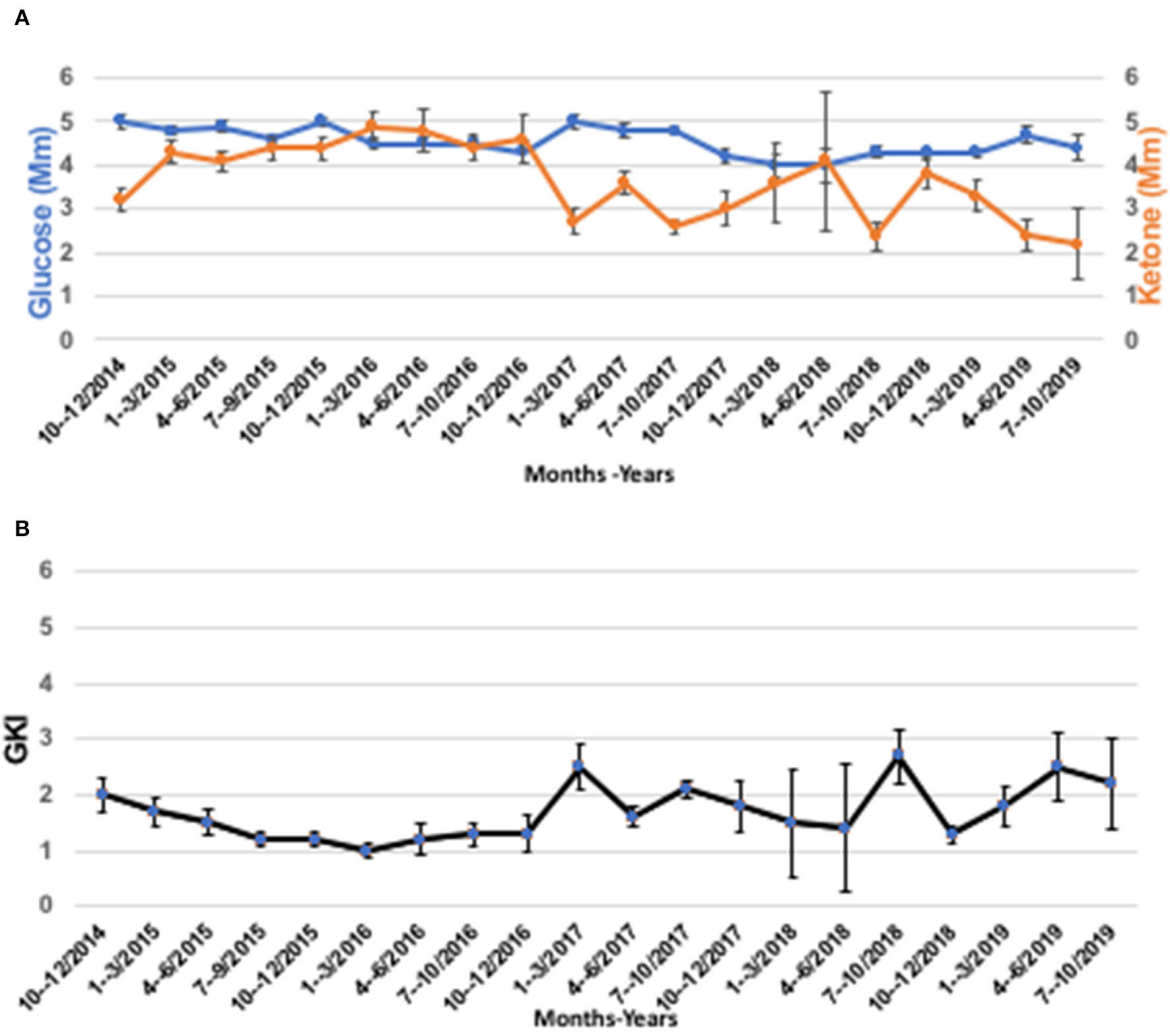
The patient's blood glucose, ketone, and GKI values from 2014 to 2019. **(A)** Blood glucose, and ketone (β-OHB) values determined using Precision Xtra blood glucose & ketone meter as described in text. **(B)** GKI values were determined from the individual glucose and ketone values in A using the glucose ketone index calculator, as previously described (77). Individual values were pooled over 3-month time intervals and are expressed as means ± 95% confidence intervals (CI). The number of readings for each data point in A and B ranged from a high of *n* = 151 (July-September, 2017), to a low of *n* = 7 (April-June 2018), and are given in the Supplementary Tables 1A–F arranged by year from 2014 to 2019.

An MRI preformed on October 9, 2018 showed interval progression of the lesion. The patient quickly realized that the regrowth of his tumor might have been linked in part to the relaxation of his dietary rigor. Along with optimization and intensification of the dietary regime, the patient adopted lifestyle interventions including moderate physical training, breathing exercises, and physiological stress management. As of November 2018, the patient has adhered to a two-meal/day schedule with a rigorous time-restricted eating regimen (20 h/day fasting). The diet consisted of eggs, bacon fried in ghee/butter (11:00 h), and steak, lamb chops, beef patties, and liver, all fried in ghee/butter/lard (16:00 h). The patient did not continue with MCT oil after he started the carnivore diet. The patient did not keep a specific food diary. When he was on a restricted calorie ketogenic diet, he would start out by weighing his food and keeping under 2,000 calories/day, but he ended up learning to judge food intake by how hungry he was and ate until he was satiated. Carbohydrates were strictly eliminated from the patient's diet. The patient recognized that a well-formulated animal-based Paleo-carnivore diet would provide most bio-available micronutrients (78). This carnivore nutritional fasting schedule returned the patient's GKI values to 2.0 or below. The patient's BMI normalized to 22.2 (72 kg) at the time of this report. In addition, the patient participated in a breathing program involving breath-holding that increased the weekly average from 15 to 60 s. and lowered the morning average resting heart rate from 80 to 60 bpm. The patient was weaned off all medications except for Zebanex (eslicarbazepine acetate, 1,200 mg) needed for seizure control, which is taken once at 16:00 h. each day. The patient's blood glucose and β-OHB values are shown in Figure 3A over an almost 5-year period, and his computed GKI values over this period are shown in Figure 3B. The raw numbers for these values are presented in the Supplementary Tables.

**Figure 4:**
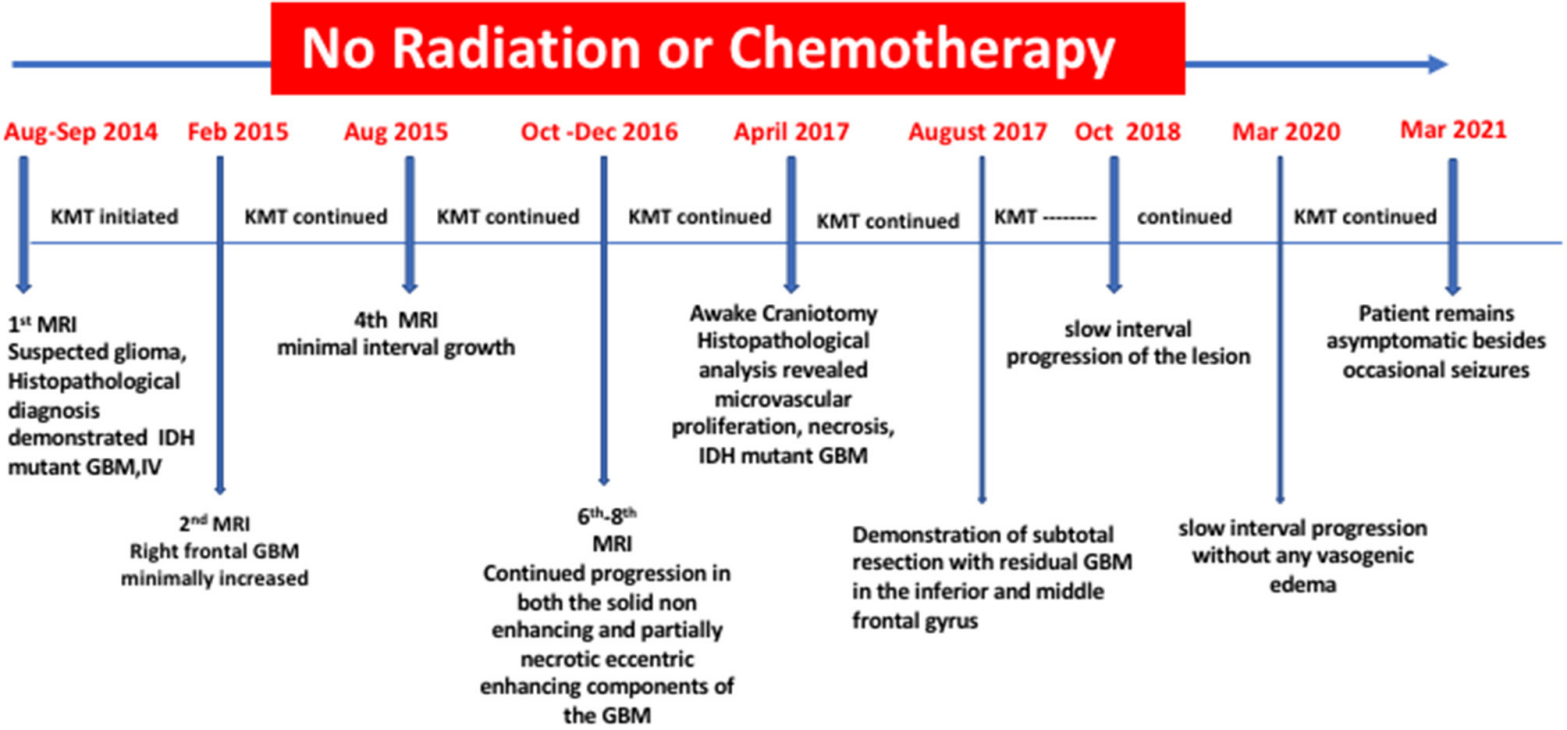
Timeline of clinical course with dates of dietary treatments and MRI.

An additional seven MRI evaluations, spanning from December 28, 2017 to March 10, 2020, showed continued slow interval progression of disease, without formation of noticeable vasogenic edema. At the time of this report (April 2021), the patient is active with a good quality of life, except for occasional tonic-clonic seizures and no signs of increased intracranial pressure. The patient was a speaker at the September 2018 Childhood and TYA Cancer Conference (http://www.childhoodcancer2018.org.uk/programme.asp; Children with Cancer; London, UK). He maintains a Facebook page that provides updates on his health status (http://www.childhoodcancer2018.org.uk/speakers/pablo-kelly.asp). Figure 4 presents a schematic diagram showing the clinical time course of MRI analysis and dietary treatment.

## Discussion

Although long-term survival is rare in patients with GBM, about 5–13% of GBM patients can survive > 5 years with SOC for reasons that are unclear (79). This case study describes long-term survival and therapeutic management with KMT in a 32-year-old man diagnosed with a histopathologic and radiographically verified *IDH1*-mutant GBM. Several factors could contribute to the long-term and continued survival of this patient (now at 80 months). First, the patient refused SOC and steroid medication. Due to his preference for non-toxic therapies and the recognized potential of KMT for GBM management, the patient opted for a self-administered KD with various supplements. This strategy, in association with the surgical debulking, could have contributed in part to the slow growth and more effective resection of his GBM. It is well-documented that survival is longer in younger GBM patients (<50 years) than in older GBM patients (> 50 years), and that patients receiving a more complete tumor resection generally survive longer than patients receiving a subtotal resection (10, 80–82). Total or subtotal neurosurgical resection, however, is generally obtained early after diagnosis to achieve a longer survival. In contrast, this patient opted for a watch and wait strategy due to his refusal of the SOC. Consequently, tumor debulking was performed almost 3 years after diagnosis. It is also known that median survival is longer in GBM patients that express the *IDH1* (*R132H*) mutation (31 months) than in patients that express the wild type allele (15 months) (13, 83). While we recognize that the therapeutic response seen in this patient might not be seen in other similarly-treated GBM patients, there are decades of compelling science supporting the mechanisms by which this metabolic therapy could reduce progression of GBM (17).

**Figure 5:**
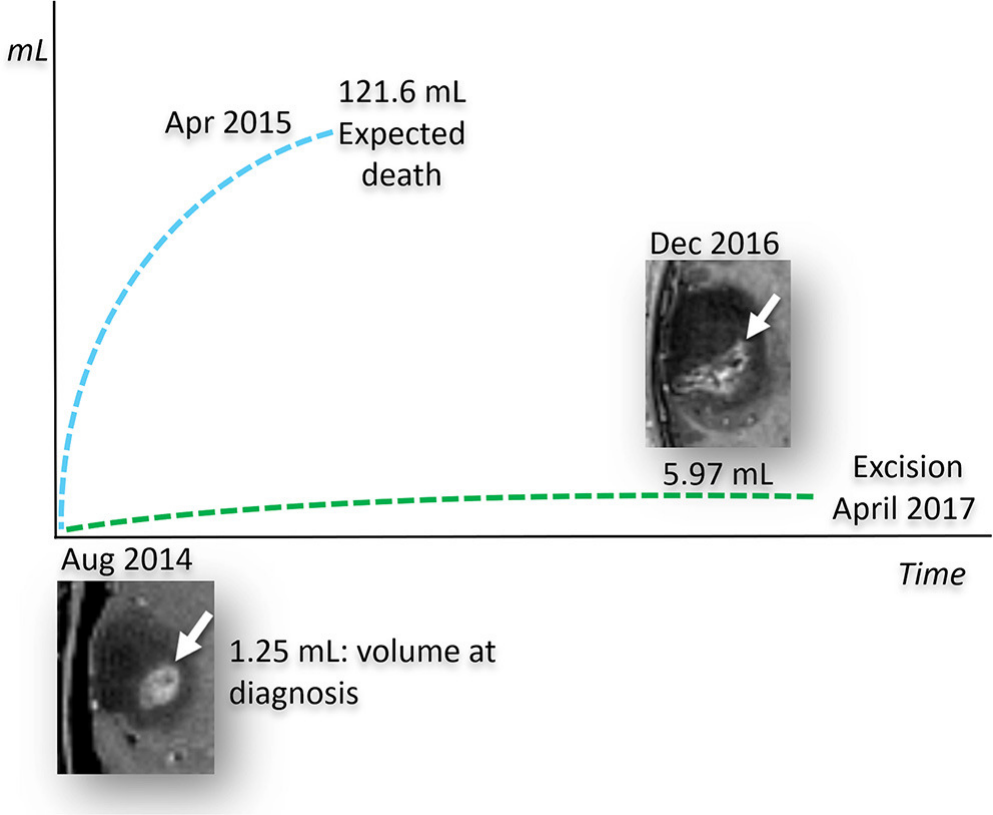
Predicted and observed tumor volume for untreated *IDH1*-mutant GBM. Measured growth of the patient's tumor compared to its estimated growth, based on the stochastic model of untreated human glioblastomas (84). The size of the tumor, measured at diagnosis in August 2014, is calculated by measuring the post contrast images only, since the current literature does not provide growth models based on the total volume of the tumor represented by the enhancing and non-enhancing tumor. Based on this stochastic model, the predicted growth rate (blue dashed line), shows that the enhancing tumor would have reached a volume incompatible with life around April 2015. In striking contrast to what was expected, the patient's *IDH1* mutant GBM, treated with the KMT alone, demonstrated a much lower growth rate (green dashed line). It should be noted that over 70% of the patient's GBM did not enhance as would expected for IDH mutation. The patient's tumor volume measurements were determined from MRI, as described in text.

The remarkably slow growth of the patient's tumor stands in contrast to previous studies on the MRI growth dynamics of untreated glioblastomas (82). Analysis of 106 untreated GBMs showed a median volume of 17.7 mL at the diagnostic MRI scan, and 27.5 mL at the preoperative scan with an estimated volume doubling time of 49.6 days. Moreover, volume doubling time was significantly faster for smaller tumors at diagnosis (<3.88 mL) than for larger tumors (> 39.88 mL). Previous studies also showed that surgical resection did not significantly increase survival in patients with small tumors no matter what percentage of the tumor could be debulked (84). GBM survival time was estimated at 292 days following immediate surgical resection and 492 days if the first surgical resection debulked 80% of the tumor (84). We used the ABC/2 formula to measure the volume change in the patient's tumor over time (85). The patient's tumor measured 1.25 mL at diagnosis (August, 2014) and grew to 5.97 mL at the time of the preoperative scan (April 2017), a 32-month time interval (Figure 5). The estimated volume doubling time for the patient's tumor was 432 days and his survival time, after resection, is now over 1,400 days. Clearly, the enhancing tumor growth rate and overall survival of this patient is markedly better than those of most previously reported cases. The patient's tumor is consistent with *IDH1* mutant GBM with a mass-like morphology > 33% of non-contrast enhancing tumor (nCET), as previously described (86). It is not likely that loss of the ATRX protein or absent MGMT methylation could have contributed to the patient's survival in light of previous information linking these markers to poor survival (87). Could the patient's chosen KMT and the chance acquisition of the *IDH1* mutation have contributed to his long-term survival with GBM?

It is well-known that GBM survival and tumor growth is linked directly to blood glucose levels, i.e., high blood glucose is associated with faster disease progression and shorter survival times (18, 41, 88–97). Glucose is the fuel for aerobic fermentation (Warburg-effect), which is a driver of most malignant cancers including GBM (26, 98). Although the patient did not keep a food diary, he was able to maintain low GKI values with intermittent fasting and his chosen low-carbohydrate food choices. The patient's ability to maintain his GKI values consistently near 2.0 and below would target the Warburg-effect thus inhibiting growth of his tumor and improving his overall survival (99). Reduced blood glucose levels will not only starve the tumor of growth metabolites through glycolysis and one-carbon metabolism, but will also down-regulate the PI3K/Akt/Hif1-1α/mTOR signaling cascades that would further inhibit dysregulated tumor cell growth (17, 58, 100–103). The low GKI values were also in the direction of predicted therapeutic success for reducing lactic acid production (17, 56, 58, 77). Reduced glucose-driven lactic acid would reduce NF-kappa-β-induced inflammation and edema in the tumor microenvironment, thus reducing tumor cell angiogenesis and invasion (17, 58, 70–72, 77, 102, 104). Reduced inflammation in the tumor microenvironment could account in part for the absence of robust vasogenic edema and the slower growth of the GBM seen in our patient, as we observed previously in KMT-treated unirradiated preclinical GBM (75). It is also important to mention that the survival of our patient was much longer than that of most other GBM patients receiving KMT following SOC (45, 54, 59, 105–108). Few of the adult patients treated with KMT in these studies, however, were able to reach or maintain the GKI values predicted to have the greatest therapeutic benefit for managing GBM (77). The avoidance of radiotherapy would also prevent liquefactive necrosis, vascular hyalinization, and rapid tumor progression, as occurred in our previous KMT-treated GBM patient that was of similar age at diagnosis (17, 56). The patient's decision to use KMT as an alternative to SOC and his ability to maintain low GKI values could have contributed in part to his long-term survival and accompanying good quality of life.

**Figure 6:**
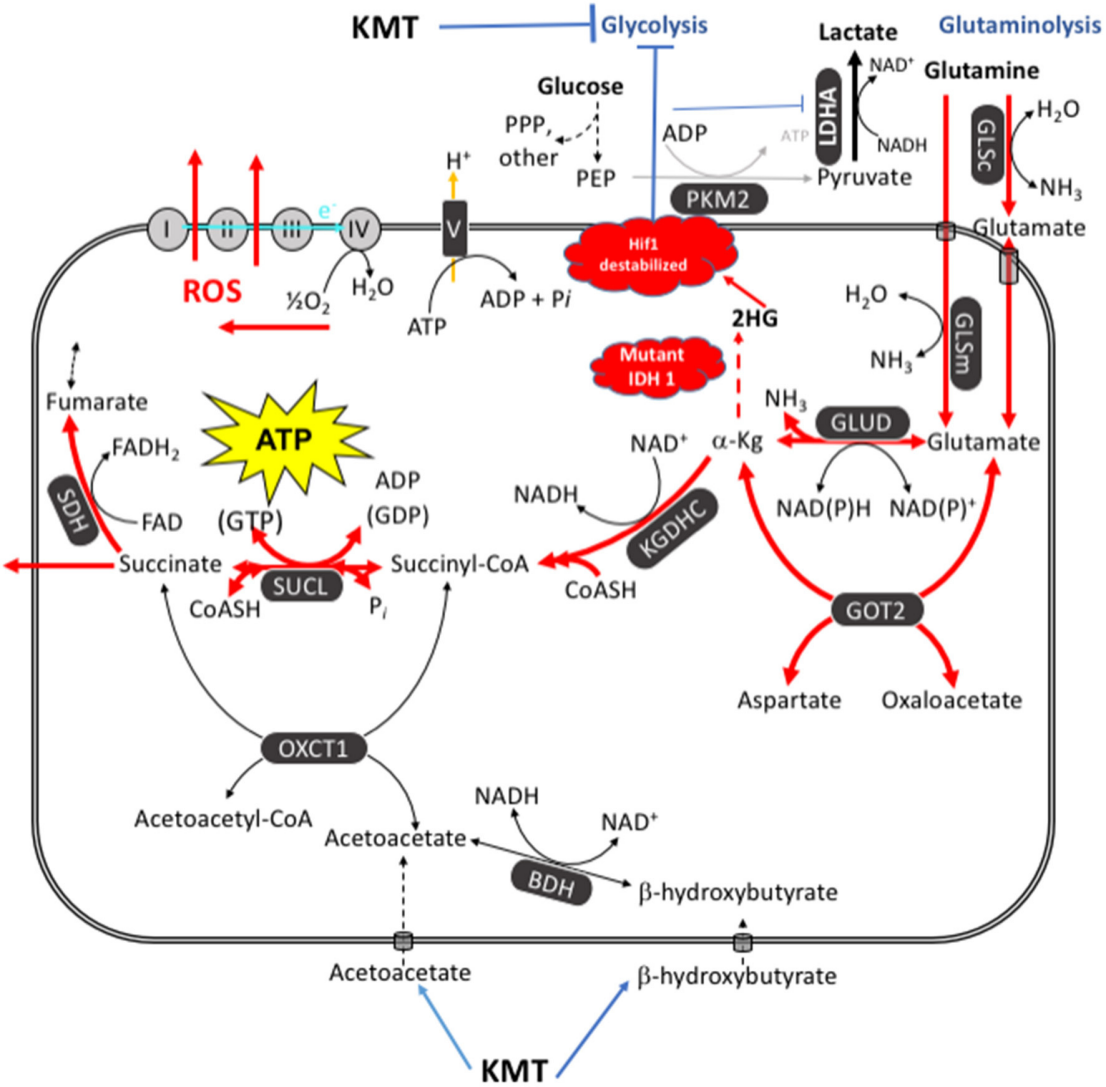
KMT/*IDH1* synergistic interaction for targeting GBM energy metabolism. KMT can reduce glucose availability for glycolysis while also interfering with the glutaminolysis pathway. Glutamine-driven mitochondrial substrate-level phosphorylation (mSLP), in the glutaminolysis pathway, is a major source of ATP synthesis for GBM cells (25, 26). The glutaminolysis pathway (red) becomes dominant in tumor cells with inefficient OxPhos and that express the dimeric PKM2 isoform. PKM2 is expressed in GBM and produces less ATP through glycolysis than does the PKM1 isoform (109–111). The elevation of ketone bodies (β-hydroxybutyrate and acetoacetate) through KMT could indirectly reduce ATP synthesis through the succinate CoA ligase (SUCL) reaction by diverting CoA from succinate to acetoacetate. The *IDH1* mutation could further reduce ATP synthesis through mSLP by increasing synthesis of 2-hydroxyglutarate from α-ketoglutarate and thus reducing the succinyl CoA substrate for the SUCL reaction (26, 112). Besides its potential effect in reducing glutaminolysis, 2-hydroxyglutarate can also target multiple HIF1α-responsive genes and enzymes in the glycolysis pathway thus limiting synthesis of metabolites and one-carbon metabolism needed for rapid tumor growth (25, 26, 103, 113). The down-regulation of Hif1-α-regulated lactate dehydrogenase A (LDHA), through the action of both KMT and the *IDH1* mutation, would reduce extracellular lactate levels thus reducing microenvironment inflammation and tumor cell invasion. Hence, the simultaneous inhibition of glycolysis and glutaminolysis through the synergistic effects of KMT and the *IDH1* mutation will stress the majority of signaling pathways necessary for rapid GBM growth. BDH, β-hydroxybutyrate dehydrogenase; FAD, flavin adenine dinucleotide; GLSc, glutaminase, cytosolic; GLSm, glutaminase; mitochondrial; GLUD, glutamate dehydrogenase; GOT2, aspartate aminotransferase; KGDHC, α-ketoglutarate dehydrogenase complex; LDHA, lactate dehydrogenase A; NME, nucleoside diphosphate kinase; OXCT1, succinyl-CoA:3-ketoacid coenzyme A transferase 1; PC, pyruvate carboxylase; PDH, pyruvate dehydrogenase; PEP, phosphoenolpyruvate; PKM2, pyruvate kinase M2; SDH, succinate dehydrogenase; SUCL, succinate-CoA ligase. Reprinted with modifications from Seyfried et al. (26).

The potential mechanism by which the *IDH1* mutation might reduce GBM growth and increase survival is discussed below and in Figure 6. We recently described how chemical energy by itself is the central issue for neoplastic cell viability. Tumors cannot grow without ATP, regardless of their cellular or genetic heterogeneity (26). In addition to glucose, glutamine is the other major fermentable fuel that can drive ATP synthesis in most cancers including GBM (24, 26, 114). Glutamine is the only amino acid that can generate ATP synthesis through mSLP in the glutaminolysis pathway (25). While KMT might not be as effective in targeting glutaminolysis as it is in targeting glycolysis, elevation of the patient's blood ketone bodies (β-hydroxybutyrate and acetoacetate), evident from his low GKI values, could indirectly target the glutamine-driven glutaminolysis pathway; also known as the Q-effect (25, 26). ATP synthesis through mitochondrial substrate-level phosphorylation (mSLP) at the succinate CoA ligase reaction (SUCL) in the glutaminolysis pathway can compensate for diminished ATP synthesis through both glycolysis and OxPhos (Figure 6). The synthesis of acetoacetyl-CoA from acetoacetate and β-hydroxybutyrate would siphon off some of the CoA needed for the synthesis of succinyl-CoA thus reducing substrate for ATP synthesis through mSLP (26). Additionally, a reduction in α-ketoglutarate levels through action of the *IDH1*-induced increase in 2-hydroxyglutarate could further reduce substrate for ATP synthesis through mSLP (25, 26, 112, 115). Recent studies also show that *IDH1*-derived 2-hydroxyglutarate can facilitate degradation of Hif1-α and thus reduce the Warburg-effect through down-regulation of multiple genes in the glycolytic pathway (113). Further evidence of an inhibitory effect of the *IDH1* mutation on glucose consumption and glycolysis was obtained recently from PET analysis (116). The long-term survival of the patient could therefore result in part from a synergistic interaction between his self-directed KMT and the anti-cancer effects of the *IDH1-R132H* mutation.

If a capability is truly important for the biology of tumors, then its inhibition should be considered as a therapeutic strategy for effective management (99, 117). The capability in this case is the fermentation metabolism needed for the synthesis of growth metabolites and ATP through the glycolytic and glutaminolysis pathways (26). As GBM “stem cells” are more dependent on glucose than on glutamine for growth, whereas GBM “mesenchymal cells” are more dependent on glutamine than on glucose, it becomes essential to inhibit both the glycolysis and the glutaminolysis pathways simultaneously to achieve maximal GBM management (24, 75). The glutamine-addicted GBM mesenchymal cells arise from neoplastic microglia/macrophages (118, 119). A recent study showed that expression of the macrophage/microglial marker, CD163, was lower in an *IDH1*-mutant GBM than in *IDH1* wild-type GBM (120). CD163 is a biomarker for glutamine-dependent neoplastic macrophages in tumor tissue (119, 121). Hence, a synergistic interaction between the effects of the *IDH1* mutation and KMT could simultaneously down-regulate both the Warburg-effect and the Q-effect in the GBM neoplastic cell populations thus providing a novel mechanism contributing to the long-term survival of our patient.

Although N-of-one single subject studies have been considered the ultimate strategy for individualizing medicine (122), we cannot predict if the therapeutic response to KMT seen in our GBM patient will also be seen in other similarly treated GBM patients, especially those with tumors that are wild-type at the *IDH1* locus. For those GBM patients not fortunate enough to have acquired the spontaneous *IDH1* mutation in their tumor, glutamine targeting drugs used with KMT will be necessary to reduce tumor growth. Our recent bench-to-bedside translational studies show that simultaneous targeting of glucose and glutamine availability, using KMT and the pan-glutaminase inhibitor, 6-diazo-5-oxo-L-norleucine (DON), can significantly prolong survival in preclinical syngeneic glioblastomas (75). It is also important to note that ketogenic diets can facilitate delivery of small-molecule therapeutic drugs through the blood brain barrier without toxicity (75, 123, 124). As GBM, like most malignant cancers, is dependent on fermentation for ATP synthesis and survival, the simultaneous restriction of fermentable fuels, i.e., glucose and glutamine, while elevating non-fermentable ketone bodies, offers a non-toxic therapeutic strategy for managing GBM. Further studies will be needed to test this hypothesis in other patients diagnosed with GBM.

## Data Availability Statement

The raw data supporting the conclusions of this article will be made available by the authors, without undue reservation.

## Ethics Statement

Written informed consent was obtained from the individual(s) for the publication of any potentially identifiable images or data included in this article.

## Author Contributions

TS: wrote the manuscript and assisted in data presentation and analysis. AS: conducted the histological report. MK: provided information on nutritional status and helped write the paper. JM: re-evaluate the patient's data and edit the paper. PM: evaluated data and assisted in manuscript preparation. GZ: analysis of MRI images and helped in writing the paper. All authors contributed to the article and approved the submitted version.

## Conflict of Interest

MK was employed by Dietary Therapies LLC. The authors declare that this study received funding from the Foundation for Metabolic Cancer Therapies, CrossFit Inc., The Nelson and Claudia Peltz Family Foundation, Lewis Topper, The John and Kathy Garcia Foundation, Edward Miller, the patient himself, the George Yu Foundation, Kenneth Rainin Foundation, Children with Cancer UK, and the Boston College Research Expense Fund. The funders were not involved in the study design, collection, analysis, interpretation of data, and the writing of this article or the decision to submit it for publication.
