# Supplementary material for: Ketogenic Metabolic Therapy, Without Chemo or Radiation, for the Long-Term Management of IDH1-Mutant Glioblastoma: An 80-Month Follow-Up Case Report
Source: Front Nutr. 2021 May 31;8:682243. doi: 10.3389/fnut.2021.682243 (PMC8200410; doi:10.3389/fnut.2021.682243)
Supplement: Supplementary file 1 [file Data_Sheet_1.PDF]

Table 1A, The patient's Glucose, Ketone, and GKI values for 2014

Date	Reading Time	Blood Glucose	Blood Ketone	GKI
10/21/14	17:40	3.6	3.7	1.0
	22:28	4.1	4.8	0.9
10/22/14	17:30	3.4	4.3	0.8
	21:22	4.9	2.7	1.8
10/23/14	15:05	4.7	4.2	1.1
10/24/14	20:33	4.6	2.7	1.7
10/25/14	14:00	4.3	2.7	1.6
	18:00	4.8	2.7	1.8
10/26/14	15:33	4.5	4.2	1.1
	22:34	4.5	4.2	1.1
10/28/14	21:30	4.4	4.1	1.1
10/29/14	21:30	4.2	3.3	1.3
10/30	8:10	4.4	1.8	2.4
	21:47	4.7	4.3	1.1
10/31/14	16:39	3.7	4.7	0.8
	22:04	4.9	4.1	1.2
11/1/14	15:45	4.6	4.2	1.1
	21:00	4.3	2.3	1.9
11/3/14	6:57	4	5.9	0.7
	22:00	4.2	5	0.8
11/4/14	0:14	4.4	5.6	0.8
11/5/14	18:17	4.2	5.3	0.8
11/6/14	20:10	4.7	2.6	1.8
11/7/14	8:00	4.3	2.4	1.8
	18:01	4.3	3.9	1.1
11/8/14	18:15	5.6	3.6	1.6
	21:24	4.4	5.9	0.7
11/9/14	15:02	4.7	3.2	1.5
	20:46	4.9	2.2	2.2
11/10/14	15:24	4.3	4.1	1.0
	21:01	4.3	5.2	0.8

11/11/14	17:40	4.3	5	0.9
11/12/14	15:52	3.8	4.1	0.9
	22:36	4.9	4.2	1.2
11/13/14	17:23	4.8	3.2	1.5
	21:54	5.3	4.4	1.2
11/14	14:49	5.8	2.8	2.1
	21:56	4.9	3.3	1.5
11/15/14	13:18	4.8	3.7	1.3
	21:59	5.2	5	1.0
11/16/14	17:06	4.5	5.6	0.8
	21:01	5.1	2.4	2.1
11/17/14	15:38	4.1	3.4	1.2
	22:26	5.2	2.9	1.8
11/18/14	18:30	5.4	1.8	3.0
	0:12	4.5	3.9	1.2
11/19/14	14:00	5.1	3	1.7
	23:15	6.1	2.1	2.9
11/20/14	13:33	4	4.3	0.9
	22:55	5.6	3.8	1.5
11/21/14	12:53	5.4	2.8	1.9
	22:25	5.1	2.6	2.0
11/26	23:15	5.2	3.3	1.6
	22:34	4.8	2.2	2.2
11/30/14	13:50	5.3	3.7	1.4
12/7/14	10:12	4.8	1.7	2.8
	17:15	5.5	3.7	1.5
	22:16	5.7	4.4	1.3
12/8/14	7:50	5.4	2.9	1.9
	15:39	4.5	4.8	0.9
	20:29	6.1	2.4	2.5
12/9/14	9:03	5	3.2	1.6
	23:25	5.4	3	1.8
12/10/14	16:49	4.8	4.4	1.1

	22:47	5.1	4.1	1.2
12/11/14	20:52	6.1	2.7	2.3
12/12/14	17:00	4.7	6.1	0.8
12/13/14	9:01	6.2	2.6	2.4
	17:55	5.7	2.9	2.0
	20:00	5.1	2.9	1.8
12/14/14	20:07	5.9	4.7	1.3
12/16/14	10:11	5.6	1.9	2.9
12/17/14	11:35	5.5	2.1	2.6
12/18/14	13:07	5.4	4.4	1.2
	14:03	4.8	4.7	1.0
12/19/14	13:00	6.9	0.9	7.7
12/20/14	18:35	5.9	1.7	3.5
	23:33	6.1	1.1	5.5
12/21/14	22:04	6.2	1.2	5.2
12/22/14	13:43	5.8	1.9	3.1
	23:05	6.4	0.8	8.0
12/23/14	15:34	4.6	2.9	1.6
	22:50	5.3	2.3	2.3
12/24/14	13:15	5.4	2.1	2.6
	23:25	6.6	2.6	2.5
12/25/14	18:34	5.6	1.6	3.5
12/26/14	9:25	5.9	0.9	6.6
	16:54	4.4	2.6	1.7
	22:34	5.3	2.8	1.9
12/27/14	7:21	5.4	0.8	6.8
	17:38	4.7	2.5	1.9
12/28/14	17:51	5.8	0.9	6.4
	22:49	5.4	1.7	3.2
12/29/14	17:37	4.9	2.9	1.7
	22:03	4.9	2.2	2.2
12/30/14	16:59	3.2	2.6	1.2
	22:02	5.3	2.3	2.3

Table 1B, The patient's Glucose, Ketone, and GKI values for 2015

Date	Time	Blood Glucose	Blood Ketone	GKI
1/1/15	17:02	5.4	1.7	3.2
	23:40	5.6	2	2.8
1/2/15	19:18	4.4	4.1	1.1
1/3/15	15:50	5.3	2.4	2.2
	22:07	5.1	2.6	2.0
1/4/15	15:26	5.3	0.4	13.3
	15:43	6.2	2.1	3.0
	21:21	6.7	1.9	3.5
1/5/15	13:26	5.6	0.8	7.0
	17:31	4.9	3.1	1.6
	21:26	5.7	2.2	2.6
1/6/15	18:18	5.3	2.8	1.9
	23:08	4.9	2.8	1.8
1/7	15:46	5.1	3.4	1.5
	21:49	4.9	1.9	2.6
1/8/15	18:35	4.8	2.7	1.8
	22:16	4.5	2.3	2.0
1/9/15	16:20	4.9	2.9	1.7
	20:54	5.4	2.5	2.2
1/10/15	17:17	4.4	3.1	1.4
	21:29	5.3	3.8	1.4
1/11/15	17:52	5	3.9	1.3
	21:10	4.7	4	1.2
1/12/15	14:17	4.9	2.8	1.8
	20:23	4.9	4.2	1.2
1/13/15	18:06	4.3	4.7	0.9
	23:14	4.7	2.9	1.6
1/14/15	17:07	4.4	4.8	0.9
	22:16	4.5	4.7	1.0
1/15/15	17:20	5	5.3	0.9

	21:57	4.9	4	1.2
1/16/15	22:01	4.7	3.4	1.4
1/17/15	21:28	4.7	4.3	1.1
1/18/15	23:26	5.3	2.3	2.3
1/19/15	18:47	3.8	4.6	0.8
	21:27	5.3	3.3	1.6
1/20/15	19:46	4.2	2.7	1.6
	22:42	5.2	3	1.7
1/21/15	16:36	4.7	2	2.4
	22:01	4.8	3.8	1.3
1/22/15	17:02	4.9	3.9	1.3
	21:11	3.9	3.8	1.0
1/23/15	17:34	4	4.2	1.0
	21:09	4.4	5.2	0.8
1/24/15	18:09	5	3.4	1.5
1/25/15	16:41	5.3	4.4	1.2
	22:41	4.9	4.1	1.2
1/26/15	18:38	5.1	2.6	2.0
	22:30	4.9	3.7	1.3
1/27/15	22:19	5	2.9	1.7
1/28/15	14:51	5.2	4.8	1.1
	21:55	4.6	3.3	1.4
1/29/15	16:52	4.8	4.2	1.1
	22:29	4.7	3.2	1.5
1/30/15	17:45	4.4	5	0.9
	22:10	5.8	2.2	2.6
1/31/15	13:00	5	2.2	2.3
	17:55	4.7	4.2	1.1
	22:01	5.4	2.7	2.0
2/1/15	13:44	4.7	2.4	2.0
	22:57	3.9	5.7	0.7
2/2/15	13:48	5.1	1.7	3.0
	17:18	4	5.1	0.8

	22:29	4.8	6.3	0.8
2/3/15	11:27	3.9	2.3	1.7
	23:18	5.1	4.3	1.2
2/4/15	17:47	5.2	3.7	1.4
	22:55	4.6	3.3	1.4
2/5/15	21:52	5.4	1.3	4.2
2/6/15	9:12	5.6	1.1	5.1
	14:28	4.2	3.7	1.1
	17:44	4.3	4.2	1.0
2/7/15	19:32	4.1	6.1	0.7
	22:33	4.4	3.9	1.1
2/14	19:12	5.4	2.3	2.3
	21:57	4.9	3.2	1.5
2/15/15	14:26	4.2	2.5	1.7
	21:12	4.9	4.1	1.2
2/16/15	17:14	5.3	3.3	1.6
2/17/15	12:15	5.7	1.1	5.2
	14:45	4.8	2.6	1.8
	21:10	5.4	1.1	4.9
2/19/15	16:36	5.1	2.8	1.8
	21:37	4.8	4.2	1.1
2/20/15	17:40	3.8	4.1	0.9
	21:55	4.9	4.7	1.0
2/21/15	16:59	5.3	4.6	1.2
	22:25	4.9	3.5	1.4
2/22/15	19:41	5.3	2.9	1.8
	21:40	4.3	3.4	1.3
2/23/15	15:35	5	3.1	1.6
2/24/15	16:56	4.7	3.5	1.3
	22:30	5	1.8	2.8
	17:28	5	2.7	1.9
2/26/15	0:02	4.7	3.9	1.2
	20:12	4.8	2.3	2.1

	23:38	4.3	2.9	1.5
2/27/15	15:38	4.8	3.3	1.5
	22:02	5.3	2.8	1.9
2/28/15	17:57	5.5	2.6	2.1
3/1/15	15:08	5.1	2.7	1.9
	21:33	4.2	3.9	1.1
3/2/15	21:41	4.7	3.2	1.5
3/3/15	22:40	4.4	2.8	1.6
3/4/15	13:43	5.1	2.8	1.8
	21:26	3.9	4.7	0.8
3/5/15	17:02	4.7	4.8	1.0
	21:59	5	2.8	1.8
3/6/15	16:54	5.2	4.6	1.1
	23:11	4.4	4.8	0.9
3/7/15	22:42	4.5	1.5	3.0
3/8/15	14:58	4.7	2.2	2.1
		5.3	4.3	1.2
3/9/15	17:38	4.2	4.8	0.9
	20:25	4.7	3	1.6
3/10/15	18:52	5.4	3/4	0.0
3/12/15	17:32	4.4	4.5	1.0
	21:37	4.4	3.4	1.3
3/13/15	16:00	4.4	2.8	1.6
3/14/15	0:18	3.8	5.1	0.7
	16:47	5.2	3.2	1.6
	21:43	4.1	5.9	0.7
3/15/15	16:41	4.4	5.1	0.9
	19:49	5.3	4.8	1.1
	22:06	4.6	4.9	0.9
3/16/15	21:29	4.5	4.8	0.9
3/30/15	16:56	3.9	3.8	1.0
4/2/15	17:32	5.4	2.9	1.9
	21:19	5.2	4.4	1.2

4/3/15	16:32	7.7	1.8	4.3
	19:40	5.4	3.6	1.5
4/4/15	15:33	5.3	1.9	2.8
	22:23	5.2	3.6	1.4
4/5/15	16:01	4.9	3.5	1.4
	22:40	4.4	4.2	1.0
4/6/15	15:40	5.9	1	5.9
	15:40	6.1	2.1	2.9
	16:12	7	0.7	10.0
	21:16	4.9	2.3	2.1
4/8/15	13:59	5.2	1.9	2.7
	16:22	5.8	1.3	4.5
	21:25	4.7	4	1.2
4/14/15	12:15	5.5	1.6	3.4
	17:52	5.9	3.8	1.6
	20:43	4.7	3.9	1.2
4/15/15	16:12	5.1	3	1.7
	23:20	3.9	5.8	0.7
4/16/15	16:29	5.1	3.4	1.5
	23:55	4.8	3.9	1.2
4/17/15	17:42	4.6	4.2	1.1
	20:46	4	6.6	0.6
4/18/15	17:23	4.3	3.8	1.1
4/19/15	18:44	5.4	2.6	2.1
4/20	16:57	4.3	3.7	1.2
4/22/15	18:12	7.7	1.9	4.1
4/23/15	19:07	5.3	2.8	1.9
4/24/15	0:33	4.2	4.1	1.0
	13:43	4	3.9	1.0
	20:11	4.5	4.6	1.0
4/25/15	19:50	4.3	4.2	1.0
4/27/15	14:44	4.8	3.7	1.3
4/28	18:08	4.3	4.7	0.9

	21:45	5.2	4.4	1.2
4/29/15	18:25	4.7	5.9	0.8
4/30/15	19:25	4	6.4	0.6
5/1/15	22:36	3.9	7.3	0.5
5/2/15	23:33	5.2	3	1.7
5/3/15	19:00	3.9	5.5	0.7
5/4/15	0:12	4.8	4.9	1.0
	18:57	4.3	5.5	0.8
5/5/15	18:54	4.3	5.1	0.8
	23:07	4.8	2.8	1.7
5/6/15	22:11	4.7	4.1	1.1
5/7/15	17:23	4.2	5.3	0.8
5/8/15	21:58	4	5.3	0.8
	23:03	4.7	6.5	0.7
5/9/15	17:20	4.5	4.6	1.0
	21:42	4.2	6.2	0.7
5/10/15	19:24	5.7	3.8	1.5
	23:54	4.8	6.3	0.8
5/11/15	16:11	4.4	4.3	1.0
	23:11	4.9	3.5	1.4
5/12/15	17:58	4.9	5.7	0.9
	22:30	5.2	4.8	1.1
5/13/15	18:08	4.4	6.1	0.7
5/14/15	21:54	4.6	4.2	1.1
5/15/15	18:03	4.7	6.4	0.7
	22:39	5.2	3.9	1.3
5/15/15	18:20	4.6	4.3	1.1
	22:42	4.3	6.3	0.7
5/17/15	16:02	4.1	3.8	1.1
	23:48	4.8	6.1	0.8
5/18/15	16:58	5.5	4.4	1.3
5/19/15	17:59	4.9	4.8	1.0
5/20/15	18:37	5.4	3.2	1.7

	22:59	5.3	3.9	1.4
5/21/15	18:02	4.7	5.4	0.9
	22:14	5.3	3.3	1.6
5/22/15	16:32	5.4	3.2	1.7
	22:20	4.5	3	1.5
5/23/15	19:07	5.6	2.1	2.7
	22:45	5.2	1.6	3.3
5/24/15	21:15	5.4	2.3	2.3
	23:25	4.7	4.5	1.0
5/25/15	16:20	5.8	2.2	2.6
	20:09	4.3	6.2	0.7
	1:33	4.9	5.7	0.9
5/26/15	18:27	4.6	6.2	0.7
5/27/15	17:48	4.4	6.8	0.6
	23:13	5.3	4.3	1.2
5/28/15	18:34	4.7	4.8	1.0
5/31/15	17:43	4.6	5.5	0.8
6/1/15	17:10	5.4	2.8	1.9
6/2/15	0:01	4.5	4.4	1.0
	17:03	4.7	4.4	1.1
6/3/15	18:12	5.4	3.1	1.7
	22:11	4.6	3.6	1.3
6/4/15	22:42	5.7	0.7	8.1
6/5/15	15:13	5.8	4.3	1.3
	1:38	5.8	3.6	1.6
6/7/15	19:28	4.4	3.4	1.3
6/8/15	20:20	5	4.8	1.0
6/9/15	15:23	5.2	2	2.6
6/10/15	0:12	4.8	5.1	0.9
	19:40	4.9	5.9	0.8
6/11/15	18:38	4	6.4	0.6
6/12/15	14:52	4.4	4.2	1.0
	19:59	4.9	3.9	1.3

6/13/15	16:39	5.8	3.6	1.6
	21:56	5.1	2.9	1.8
6/14/15	14:10	4.5	3.6	1.3
	23:05	5.4	3.3	1.6
6/15/15	20:50	3.9	5.2	0.8
6/18/15	17:32	4.6	4.5	1.0
6/19/15	0:23	4.9	3.4	1.4
6/20/15	19:12	4.9	2.5	2.0
	23:11	4.4	4.8	0.9
6/21/15	18:27	4.2	4.4	1.0
	23:56	3.9	4.1	1.0
6/22/15	21:36	5	4.1	1.2
6/23/15	21:56:00	3.6	4.7	0.8
6/24/15	0:20	3.9	4.6	0.8
	16:42	4.9	5.6	0.9
	22:18	4.1	5.7	0.7
6/25/15	17:55	3.8	4.7	0.8
6/26/15	0:20	4.6	5.9	0.8
	20:45	4.5	4.7	1.0
6/27/15	19:28	4.4	3.7	1.2
	22:55	5.3	2.9	1.8
6/28/15	16:39	5.4	2.9	1.9
	19:45	4.1	5.7	0.7
	23:51	4.9	4.2	1.2
6/30/15	20:36	4.3	4.1	1.0
7/1/15	0:01	5.7	2.5	2.3
	20:04	4.8	2.5	1.9
	23:02	4.2	3.3	1.3
7/2/15	18:46	4	5.11	0.8
7/3/15	0:22	5.2	2.4	2.2
	16:07	4.4	5.6	0.8
	21:54	4.3	4.9	0.9
7/4/15	19:15	3.9	6.4	0.6

	23:25	3.7	6.2	0.6
7/5/15	21:40	4.9	2.3	2.1
7/6/15	19:39	3.9	4.4	0.9
7/7/15	0:27	4.1	3.5	1.2
	16:48	5.1	2.2	2.3
	22:54	4.7	5.6	0.8
7/8/15	23:34	4.2	4.6	0.9
7/9/15	20:13	4.3	5.2	0.8
	23:33	4.6	3.6	1.3
7/10/15	16:10	4.7	5.4	0.9
	23:22	4.8	3.8	1.3
7/12/15	17:41	5.1	4.2	1.2
7/15/15	8:41	5.2	1.6	3.3
	23:12	4.7	2.4	2.0
7/16/15	18:42	4.2	5.9	0.7
7/18/15	19:07	4.1	5.8	0.7
7/20/15	21:31	3.3	7.7	0.4
7/21/15	21:11	4.1	4.2	1.0
7/22/15	23:29	4.3	4.7	0.9
7/23/15	22:47	5.1	2.9	1.8
7/24/15	19:40	4.4	4.4	1.0
	23:39	4.7	5.8	0.8
7/25/15	19:52	4.1	4.3	1.0
	23:36	5.2	6	0.9
7/26/15	19:04	3.3	5.5	0.6
7/27/15	23:45	4.6	4.1	1.1
7/29/15	19:46	4.4	6.3	0.7
	23:44	5	4.7	1.1
7/30	23:49	3.7	5.3	0.7
7/31/15	0:13	4.9	3.3	1.5
8/3/15	23:37	4.9	4.2	1.2
8/4/15	19:35	4.5	7.1	0.6
8/5/15	0:32	4.2	4.6	0.9

	19:12	4.7	2.8	1.7
8/7/15	15:47	4.1	6.6	0.6
	10:01	4.4	4.7	0.9
8/9/15	19:25:00	3.3	4.1	0.8
	23:11	4.7	5.5	0.9
8/11/15	21:21	4.1	6	0.7
8/14/15	22:37	5.1	5.1	1.0
8/15/15	0:19	4.9	3.9	1.3
8/19/15	0:21	5	5.4	0.9
8/20/15	22:37	5.1	3.1	1.6
8/21/15	23:05	4.6	4.4	1.0
8/25/15	21:15	4.8	3.7	1.3
8/26/15	21:56	5.1	3.1	1.6
8/31/15	21:57	4.5	3.8	1.2
9/1/15	20:53	4.4	5.3	0.8
9/2/15	21:54	4.4	4.3	1.0
9/3/15	22:22	4.9	5.7	0.9
9/5/15	23:14	5	4.2	1.2
9/6/15	22:06	5.1	3.5	1.5
9/7/15	18:22	4.2	5	0.8
	23:05	4.6	5.7	0.8
9/8/15	16:20	4.9	4.3	1.1
	22:32	5.6	2.1	2.7
9/9/15	20:08	4.2	6.6	0.6
	22:53	5.1	4.8	1.1
9/10/15	20:10	4.2	6	0.7
9/11/15	8:46	6.3	5.7	1.1
	20:22	4	4.7	0.9
	23:47	4.6	5.8	0.8
9/12/15	15:56	4.3	5.7	0.8
9/15/15	14:36	6.3	1.8	3.5
	17:16	4.5	3.1	1.5
	23:37	5.2	4.4	1.2

9/16/15	17:35	4.6	5.3	0.9
	23:36	4.6	4.1	1.1
9/17/15	22:02	4.5	4.6	1.0
9/18/15	19:40	4.6	3.6	1.3
	22:53	5.1	4	1.3
9/19/15	19:53	4.3	3.8	1.1
	22:39	5.3	3.2	1.7
9/21/15	16:58	5.1	4.6	1.1
9/22/15	16:56	5.5	2.8	2.0
	23:32	4.6	3.7	1.2
9/23/15	19:33	4.6	5	0.9
9/25/15	17:23	4.9	2.8	1.8
	22:59	5.3	4.1	1.3
9/26/15	17:25	4.3	4.7	0.9
	22:02	5.2	3.4	1.5
9/27/15	23:43	5.7	3	1.9
9/30/15	23:53	4.9	3.7	1.3
10/1/15	16:11	5.6	3.8	1.5
10/3/15	23:34	5.3	3.6	1.5
10/5/15	19:05	4.3	6.5	0.7
10/6/15	18:39	4.5	6.5	0.7
10/7/15	0:50	4.2	5.9	0.7
	20:27	4.4	5.5	0.8
	23:54	5.6	3.7	1.5
10/8/15	0:43	5.1	3.2	1.6
10/9/15	19:41	4.7	5.5	0.9
10/14/15	20:17	5.1	3.4	1.5
10/15/15	16:38	4.8	4.9	1.0
	23:41	5.6	2.4	2.3
10/16/15	0:06	5.6	2.5	2.2
10/17/15	23:13	4.6	2.6	1.8
10/18/15	20:37	4.1	4.3	1.0
10/19/15	20:33	4.4	5.6	0.8

10/20/15	16:44	4.8	5.1	0.9
	20:19	4.1	5.8	0.7
	23:50	5.7	2.4	2.4
10/22/15	0:54	4.8	5.1	0.9
10/23/15	18:50	4.6	5.8	0.8
10/24/15	1:21	5.1	4.1	1.2
10/25	17:22	4.6	4.1	1.1
10/26/15	19:30	5.2	4.4	1.2
10/27/15	21:02	4.7	4.6	1.0
	23:52	4.8	3.2	1.5
10/29/15	19:25	4.9	4.9	1.0
10/30/15	16:25	5.6	2.4	2.3
		5.4	2.1	2.6
10/31/15	23:59	4.2	3.8	1.1
11/4/15	23:34	5.8	2.6	2.2
11/5/15	21:08	5.3	5.2	1.0
		4.9	3.9	1.3
11/7/15	23:41	5.2	3.6	1.4
11/8/15	20:05	4.7	5.7	0.8
	21:55	5.2	2.2	2.4
11/9/15	16:39	4.4	5.6	0.8
	20:00	4.4	5.6	0.8
11/10/15	21:02	4	5.7	0.7
	23:26	4.1	3.4	1.2
11/7/15	17:39	5.4	4.7	1.1
	21:39	4.7	3.9	1.2
	1:06	4.3	5.4	0.8
11/16/15	14:04	4.6	4.7	1.0
	20:00	4.7	5.8	0.8
	23:43	5.4	2.8	1.9
11/17/15	23:20	6.6	4.2	1.6
11/19/15	18:07	4.7	5.5	0.9
11/20/15	21:59	5.2	3.8	1.4

11/21/15	16:01	4.9	4.5	1.1
	23:15	5.1	3.1	1.6
11/22/15	21:12	4.1	5.7	0.7
	22:49	6.1	3.1	2.0
	23:54	5.3	3.2	1.7
11/23/15	17:45	4.7	5.1	0.9
	23:30	5	4.8	1.0
11/24/15	23:59	5.4	5.6	1.0
11/25/15	20:22	4.5	5.9	0.8
11/26/15	18:53	4.5	5.7	0.8
	23:51	5.6	4.1	1.4
11/27/15	20:06	4.9	4.9	1.0
11/28/15	19:33	5.1	3.2	1.6
11/29/15	18:18	4.4	4.6	1.0
	22:00	4.7	5.5	0.9
11/30/15	21:36	4.6	3.7	1.2
	23:34	6.1	3.1	2.0
12/1/15	14:39	5.4	4.2	1.3
	17:45	6.2	4.9	1.3
12/2/15	19:29	4.5	4.7	1.0
	23:39	5.8	2.7	2.1
12/3/15	22:58	6.2	2.4	2.6
12/4/15	15:38	4.7	6.1	0.8
	19:59	5.4	4.5	1.2
12/5/15	13:58	4.9	4.2	1.2
	20:07	4.9	4.7	1.0
12/6/15	18:14	4.3	5.8	0.7
12/8/15	18:32	5.5	3.1	1.8
	20:05	5.5	5.1	1.1
12/11/15	18:38	4.1	6.1	0.7
12/13/15	0:07	4.8	4.8	1.0
	16:42	5.7	5.7	1.0
12/19/15	18:54	5.3	5.7	0.9

12/26/15	17:17	4.3	5.4	0.8
12/27/15	19:14	5.2	4.6	1.1
12/30/15	20:08	4.3	5.2	0.8

Table 1C, The patient's Glucose, Ketone, and GKI values for 2016

Date	Reading Time	Blood Glucose	Blood Ketone	GKI
1/1/16	20:08	4.3	5.2	0.8
1/2/16	20:49	3.9	5.4	0.7
1/14/16	17:53	4.3	6	0.7
1/17/16	17:51	5	3.9	1.3
	21:17	4.9	4.6	1.1
1/18/16	19:06	4.9	3.9	1.3
	22:00	4.9	2.4	2.0
1/20/16	19:08	4.8	4.2	1.1
1/21/16	21:49	4.8	6.2	0.8
1/22/16	19:15	4.1	5.1	0.8
1/22/16	18:15	4.4	6.6	0.7
1/25/16	19:21	4.1	4.2	1.0
1/28/16	21:42	5.2	3.2	1.6
1/29/16	19:01	3.8	6.1	0.6
	22:41	4.6	4.6	1.0
1/30/16	18:49	3.9	4.7	0.8
1/31/16	21:08	4.6	4.8	1.0
2/1/16	18:05	4.7	5.2	0.9
2/2/16	19:09	4.4	4.4	1.0
2/3/16	19:21	4.3	3.6	1.2
2/4/16	18:38	4.1	6.2	0.7
2/5/16	19:30	4.8	7.3	0.7
2/6/16	18:06	3.8	6.6	0.6
2/7/16	18:23	4.5	5.1	0.9
2/8/16	19:46	5.5	4.5	1.2
2/9/16	19:44	4.1	6.2	0.7

2/12/16	22:59	5.1	4.7	1.1
2/13/16	22:11	4.8	5.4	0.9
2/14/16	21:25	4.4	5.2	0.8
2/15/16	21:36	4.6	4.1	1.1
2/16/16	21:00	3.9	5.3	0.7
2/17/16	21:24	3.9	4.9	0.8
2/18/16	18:15	3.5	6	0.6
2/19/16	21:24	4.4	4.2	1.0
2/20/16	18:34	4.6	3.5	1.3
2/21/16	21:02	4.7	3.5	1.3
2/22/16	18:01	4.3	5.2	0.8
2/23/16	19:20	3.9	6.2	0.6
2/25/16	19:19	4.3	4.4	1.0
2/26/16	20:33	4.3	5.1	0.8
3/8/16	18:04	4.9	3.8	1.3
3/9/16	19:43	4.1	5.7	0.7
3/13/16	18:47	4.8	5.3	0.9
3/30/16	20:08	4.4	4.8	0.9
3/31/16	15:44	5.7	2.3	2.5
	20:30	4.8	5.5	0.9
4/1/16	20:25	3.9	5.3	0.7
4/3/16	21:36	4.7	3.7	1.3
4/9/16	18:26	3.3	6.4	0.5
4/13/16	15:20	4.3	2.4	1.8
	17:30	4.6	4.8	1.0
4/14/16	19:04	4.3	5.4	0.8
4/16/16	10:09	5.7	2.6	2.2
4/18/16	19:30	4.7	3.6	1.3
4/20/16	20:19	5.6	6.8	0.8
4/21/16	18:46	5.4	1	5.4
		4.7	2.6	1.8
4/24/16	19:39	3.5	4.6	0.8
4/25/16	18:35	5.2	2.3	2.3

4/26/16	18:12	4.1	5.8	0.7
4/27/16	18:55	4.6	4.7	1.0
4/28/16	20:54	4.4	4.4	1.0
4/30/16	18:00	4.6	5.3	0.9
5/1/16	19:10	3.9	5.8	0.7
	24:00:00	4.8	5.3	0.9
5/2/16	19:19	3.9	6.1	0.6
5/3/16	17:35	4.2	3.8	1.1
5/4/16	19:04	5.3	1.4	3.8
5/5/16	19:23	4.6	3.6	1.3
	22:18	5.6	1.8	3.1
5/6/16	20:09	4.4	5	0.9
5/7/16	19:14	4.9	3.1	1.6
5/8/16	19:39	4.9	3	1.6
5/9/16	18:55	3.7	6.3	0.6
5/13/16	19:32	4.8	5.6	0.9
5/15/16	19:31	4.3	5.6	0.8
5/16/16	20:10	4.7	4.8	1.0
5/17/16	18:03	4.2	6.3	0.7
5/18/16	20:50	4.3	6.4	0.7
5/20/16	19:57	4.1	5.6	0.7
5/22/16	18:19	4.5	5.8	0.8
5/26/16	20:36	4.5	5.7	0.8
5/28/16	20:49	4.3	6.7	0.6
6/10/16	19:36	3.6	5.6	0.6
6/12/16	19:20	4.2	5.5	0.8
6/14/16	19:27	4.1	6.7	0.6
6/16/16	17:22	4.8	3.9	1.2
6/19/16	20:56	4.3	5.4	0.8
6/23/16	20:34	4	7.3	0.5
6/24/16	20:20	4.1	7.3	0.6
7/8/16	18:52	4.7	6.2	0.8
7/9/16	22:06	4.1	5.6	0.7

7/12/16	23:05	5.9	2.3	2.6
7/13/16	10:14	5.7	0.9	6.3
7/14/16	20:34	4.7	4.4	1.1
7/15/16	22:22	4.7	4.3	1.1
7/17/16	17:41	5.6	1.7	3.3
	19:16	4.7	2.2	2.1
	22:29	5.6	2.8	2.0
7/18/16	10:03	5.9	1	5.9
	12:22	5.6	2.8	2.0
	18:04	4.6	5	0.9
7/19/16	23:20	4.2	5.1	0.8
7/20/16	17:57	3.9	6.1	0.6
	22:12	4.2	5.4	0.8
7/23/16	19:30	4.7	6.6	0.7
7/24/16	20:38	4.7	6.6	0.7
7/25/16	22:26	4.6	5.4	0.9
7/26/16	20:18	4.1	6.4	0.6
7/27/16	17:36	4.7	4.4	1.1
8/1/16	19:46	4.6	4.6	1.0
8/2/16	18:49	5.2	2.9	1.8
8/3/16	20:50	4.4	5.7	0.8
8/5/16	20:33	4.1	5.3	0.8
8/9/16	12:43	4.2	5.2	0.8
8/11/16	10:09	6.4	1.2	5.3
	17:12	5.4	2.2	2.5
	19:12	4.5	4.3	1.0
8/12/16	0:35	5	4.6	1.1
	8:40	4.8	2.3	2.1
	13:28	3.8	4.4	0.9
	18:40	4.3	5.6	0.8
	23:16	5	5.2	1.0
8/13/16	10:42	5.6	2	2.8
8/14/16	15:36	4.6	4.3	1.1

	22:44	4.6	5.2	0.9
	9:49	5.5	1.8	3.1
	15:00	4.5	5.4	0.8
	20:49	4.5	5.9	0.8
8/15/16	8:07	4.9	1.8	2.7
	12:06	4.7	3.4	1.4
8/16/16	7:43	4.6	2.2	2.1
8/17/16	9:31	4.7	2.3	2.0
	19:32	5	6.1	0.8
8/18/16	10:07	5.5	2.1	2.6
	14:08	3.9	4.7	0.8
8/19/16	9:39	5.2	2.8	1.9
	19:04	4	5.2	0.8
8/20/16	9:24	4.6	2.3	2.0
	13:39	4.6	2.8	1.6
8/24/16	19:36	4.4	5.4	0.8
	22:14	4.6	5	0.9
8/25/16	9:30	5.1	3.1	1.6
	18:08	4.3	5.9	0.7
8/26/16	9:33	5.3	1.6	3.3
	14:45	4	5.8	0.7
	18:33	3.6	6.7	0.5
	21:04	4.3	3.9	1.1
8/27/16	10:15	4.5	2.6	1.7
	13:51	4.8	2.9	1.7
	16:48	5	4.2	1.2
	21:12	4	5.8	0.7
8/28/16	9:54	4.9	1.7	2.9
	17:22	4.4	4.5	1.0
8/29/16	10:00	5.6	2.8	2.0
	17:41	4.3	6.3	0.7
	21:40	4.1	5.7	0.7
8/30/16	9:44	4.7	2.5	1.9

	16:43	4.6	4.4	1.0
	21:31	4.7	5.6	0.8
8/31/16	13:52	4.9	4.3	1.1
	17:34	4.3	5.8	0.7
	21:33	5.1	3.9	1.3
9/1/16	19:49	4.1	5.3	0.8
9/1/16	14:08	4.2	4.8	0.9
	18:33	4.5	5.2	0.9
	22:10	4.5	2.1	2.1
9/3/16	18:46	3.9	4.8	0.8
9/4/16	18:42	4	5.9	0.7
9/5/16	17:45	4.9	3	1.6
	20:49	5.3	2.9	1.8
9/6/16	17:13	4.8	4	1.2
9/7/16	17:52	4.7	4.3	1.1
9/8/16	17:55	4.1	4.8	0.9
9/9/16	19:15	3.4	5.6	0.6
9/10/16	15:06	3.6	5.1	0.7
	22:09	3.9	4.9	0.8
9/11/16	18:42	3.2	6.2	0.5
9/12/16	18:11	3.7	6.2	0.6
9/13/16	16:53	4.2	5.5	0.8
	22:06	4.2	4.1	1.0
9/14/16		5	3	1.7
9/15/16	18:24	3.2	6.6	0.5
9/17/16	23:08	4.2	3.6	1.2
9/18/16	19:08	3.8	4.7	0.8
9/19/16	21:43	3.4	5.1	0.7
9/20/16	18:18	3.8	5.1	0.7
	22:00	4.6	3.3	1.4
9/22/16	20:03	3.3	7.1	0.5
9/24/16	20:11	4.1	5.5	0.7
9/25/16	19:38	3.4	6.1	0.6

9/26/16	18:46	3.9	6.6	0.6
9/27/16	17:36	4	5.9	0.7
9/29/16	20:25	3.2	7	0.5
9/30/16	17:30	3.5	6.7	0.5
10/3/16	17:21	3.6	6.5	0.6
10/4/16	17:23	3.3	6.9	0.5
10/7/16	19:54	3.9	5.8	0.7
10/12/16	17:00	4.1	5.7	0.7
10/17/16	22:08	5.4	3	1.8
10/18/16	18:24	3.6	6.1	0.6
10/20/16	18:29	4.2	5.6	0.8
10/22/16	19	3.3	6.2	0.5
10/23/16	16:09	6.6	2.2	3.0
	18:30	4.4	5	0.9
10/24/16	19:05	3.6	6.5	0.6
10/25/16	23:39	4.2	4.2	1.0
10/26/16	20:07	4.3	5.1	0.8
10/27/16	19:06	3.9	6	0.7
10/29/16	10:59	4.7	3.9	1.2
10/30/16	13:33	4.1	5.3	0.8
11/1/16	16:14	4.3	5	0.9
11/2/16	18:58	3.3	4.7	0.7
11/3/16	16:35	4.9	5.2	0.9
11/4/16	17:52	4.9	2.7	1.8
11/5/16	18:55	4.1	6.4	0.6
11/9/16	19:01	3.9	5.3	0.7
11/13/16	12:20	4.7	3.8	1.2
	19:00	3.7	6.6	0.6
11/25/16	19:13	3.4	5.2	0.7
12/3/16	19:00	3.8	5.8	0.7
12/7/16	22:11	5.9	5	1.2
12/8/16	14:53	6.2	1.6	3.9
12/9/16	9:45	4.7	1.6	2.9

	19:22	4.2	4.1	1.0
	22:52	4.7	3.4	1.4
12/11/16	9:55	4.6	2	2.3
12/12/16	9:43	4.8	1.6	3.0
	23:19	4.1	5.3	0.8
12/13/16	9:31	5.1	1.2	4.3
12/16/16	13:47	3.9	4.5	0.9

Table 1D, The Patient's Glucose, Ketone, and GKI Values for 2017

Date	Reading Time	Blood Glucose	Blood Ketone	GKI
1/4/17	19:24	3.9	4.5	0.9
1/14/17	18:34	3.9	5.7	0.7
1/15/17	18:01	3.9	4.1	1.0
2/4/17		4.6	2.1	2.2
2/9/17	22:07	4.9	1.9	2.6
2/11/17	16:14	4.4	3.2	1.4
2/15/17	21:40	5.4	2.9	1.9
2/16/17	8:26	5.2	1.1	4.7
	16:16	4.9	1.9	2.6
	17:24	4.3	1.8	2.4
	18:44	4.6	2	2.3
	21:29	4.5	4.2	1.1
2/17/17	15:13	5	3.4	1.5
	18:36	4.2	5.6	0.8
2/18/17	18:25	3.7	3.8	1.0
2/19/17	17:53	4.4	3.1	1.4
3/3/17	22:41	5.1	1.7	3.0
	23:42	5.3	1.2	4.4
3/4/17	0:11	5	1.2	4.2
	8:41	5	0.4	12.5
	13:27	5	1.4	3.6
	16:35	5.1	2.1	2.4

	19:16	4.5	4.2	1.1
3/4/17	9:27	5.2	3	1.7
	12:50	5.6	2.8	2.0
	14:40	5.1	2	2.6
3/5/17	17:04	5.2	4.4	1.2
	22:47	5.5	2.9	1.9
3/6/17	8:48	5.6	1	5.6
	11:01	5.2	1.3	4.0
	12:58	4.6	3.5	1.3
	17:34	5.4	4.3	1.3
	21:27	5.9	1.9	3.1
	23:09	6.2	2.3	2.7
3/9/17	12:00	5.7	1	5.7
	17:02	5.5	2.3	2.4
3/10/17	12:26	5.8	1.3	4.5
	18:49	6.1	2.2	2.8
3/11/17	15:55	6.4	1.4	4.6
3/12/17	18:22	6.8	2.6	2.6
3/13/17	21:55	5.5	2.6	2.1
3/14/17	15:06	5.9	2.3	2.6
3/15/17	17:39	5.6	4.4	1.3
3/16/17	16:52	5.5	1.6	3.4
	18:14	4.9	2.6	1.9
3/18/17	17:34	4.3	3.5	1.2
3/21/17	12:03	5	2.8	1.8
3/23/17	23:14	4	4.8	0.8
3/24/17	20:28	5.3	2.9	1.8
	23:58	4.1	5.2	0.8
3/25/17	11:33	5.3	2.6	2.0
	18:04	4.4	4.6	1.0
	22:03	5.2	2.6	2.0
	23:00	5.7	3.1	1.8
3/26/17	22:11	5.6	1.9	2.9

3/27/17	10:16	4.8	1.9	2.5
	19:25	4.2	4.1	1.0
	21:42	6	1.9	3.2
3/28/17	0:41	4.7	4.1	1.1
	9:00	5.2	1.6	3.3
	13:49	5.1	2.6	2.0
	17:38	4.6	4	1.2
	22:15	5.7	0.9	6.3
3/28/17	0:43	5.3	2.8	1.9
	17:48	5.2	1.7	3.1
	21:16	4.3	3.3	1.3
3/30/17	18:32	4.3	2.4	1.8
	21:24	5.1	2.2	2.3
3/31/17	14:15	5.6	1.4	4.0
	15:07	5.3	2.2	2.4
	22:30	3.8	4.2	0.9
4/5/17	20:22	4.6	3.6	1.3
4/7/17	18:33	4.2	5.1	0.8
4/8/17	17:12	5.3	2.7	2.0
	22:33	5.4	2.5	2.2
4/9/17	8:14	5.8	1.2	4.8
	16:33	5.8	4.3	1.3
4/10/17	16:04	5.3	3.6	1.5
	17:37	5.2	1.8	2.9
4/12/17	17:32	4.3	3.8	1.1
	21:48	5.4	3.3	1.6
4/13/17	16:34	5.3	3.9	1.4
4/14/17	18:38	5	3.6	1.4
4/16/17	18:50	4	4.4	0.9
	22:21	5.4	1.9	2.8
4/17/17	17:17	4.7	4.1	1.1
	20:38	4.3	3.6	1.2
4/18/17	17:32	4.6	4.7	1.0

	19:42	4.1	4.2	1.0
4/20/17	15:24	5.2	2.9	1.8
4/21/17	19:13	4.8	3.9	1.2
4/22/17	19:10	4.8	3.9	1.2
4/23/17	19:10	4.4	5.5	0.8
4/24/17	17:28	4.7	4.1	1.1
4/25/17	17:32	4.4	4.4	1.0
4/27/17	22:41	5.3	4.4	1.2
4/29/17	17:57	4	3.7	1.1
5/3/17	23:02	4.8	2.7	1.8
5/4/17	20:20	4.5	3.8	1.2
5/9/17	21:20	4.9	3.1	1.6
5/10/17	17:40	4.4	4.2	1.0
5/11/17	21:44	4.8	1.7	2.8
5/12/17	20:15	4	5.3	0.8
5/13/17	19:09	3.9	5.7	0.7
5/14/17	18:52	4.3	3.4	1.3
5/15/17	17:14	5	4.3	1.2
	23:33	1.39	1.9	0.7
	23:34	5.2	1	5.2
5/16/17	22:42	5	2.2	2.3
5/17/17	20:10	4.5	3.3	1.4
5/18/17	19:59	4.7	4.3	1.1
5/19/17	20:16	3.9	5.6	0.7
	22:55	5.3	3.4	1.6
5/20/17	18:20	4.2	5.2	0.8
5/21/17	16:35	5.2	1.9	2.7
	19:49	5.2	4	1.3
	0:06	6	2.8	2.1
	4:55	4.7	4.8	1.0
5/22/17	19:18	5.1	2.6	2.0
	22:00	4.7	3	1.6
5/23/17	17:17	5.7	2.6	2.2

	22:46	5.3	2.2	2.4
	23:42	6.7	1.8	3.7
5/26/17	20:30	4.1	4.1	1.0
5/27/17	0:01	5.5	3.2	1.7
	19:17	4.4	4.9	0.9
5/28/17	18:03	5.2	3.5	1.5
	23:03	5.7	3.8	1.5
5/30/17	23:25	4.1	4.4	0.9
5/31/17	18:17	5.5	4.3	1.3
6/1/17	21:33	4.3	5.2	0.8
6/2/17	18:35	5.6	2.8	2.0
6/3/17	20:00	4.9	4.1	1.2
6/6/17	19:48	4.3	3.7	1.2
6/7/17	18:30	5.3	2.9	1.8
6/9/17	19:12	5	2.5	2.0
6/15/17	19:28	4.5	5.3	0.8
6/16/17	0:58	6.3	1.9	3.3
	20:23	4.3	5.1	0.8
	22:53	5.1	3.7	1.4
6/17/17	19:35	4.9	3.1	1.6
6/18/17	22:10	5.3	3.6	1.5
	0:11	5.9	3.2	1.8
6/19/17	0:58	4.6	4.1	1.1
	19:49	4.4	4.9	0.9
6/21/17	23:59	4.7	3.2	1.5
6/22/17	1:19	5	3.3	1.5
6/23/17	20:03	4.7	3.9	1.2
6/24/17	16:46	4.9	3.2	1.5
6/26/17	20:32	4.2	5.1	0.8
6/29/17	20:08	5.1	4.2	1.2
6/30/17	20:01	5.1	2.1	2.4
7/1/17	4:30	6.5	2.3	2.8
	22:56	4.9	3.3	1.5

7/2/17	1:43	4.6	3.9	1.2
	3:21	6.2	1.7	3.6
	23:15	4.7	2.7	1.7
	22:13	4.4	5.2	0.8
	23:54	4.4	3.9	1.1
7/3/17	19:01	4.3	4.4	1.0
	1:32	4.4	2.8	1.6
7/4/17	23:49	4.9	2.5	2.0
7/5/17	1:11	4.3	3.1	1.4
	23:29	5.4	2.1	2.6
7/6/17	0:50	4.7	3	1.6
	20:02	4.7	2.8	1.7
	23:51	4.9	5.3	0.9
7/7/17	4:07	4.9	4	1.2
	21:52	5	5	1.0
7/8/17	22:43	4.3	4.7	0.9
7/9/17	0:55	5.3	2.8	1.9
7/10/17	1:25	1.8	2.4	0.8
	18:41	5.8	2.9	2.0
7/12/17	1:28	5.2	3.3	1.6
7/13/17	1:27	4.7	3.2	1.5
	21:08	4.5	3.2	1.4
7/14/17	2:02	4.9	3.2	1.5
	7:32	7.2	2.1	3.4
7/15/17	1:51	4.8	1.9	2.5
	2:55	6.4	2.1	3.0
7/16/17	1:39	5.3	2.5	2.1
	23:36	5.2	3.4	1.5
7/17/17	1:35	4.7	3.3	1.4
7/18/17	20:10	5.6	2.9	1.9
7/19/17	0:28	5.3	2.6	2.0
	3:19	5.4	1.3	4.2
	19:45	5.4	2.3	2.3

7/20/17	1:07	4.9	1.4	3.5
	3:13	4.8	1.6	3.0
	4:29	5.1	2.9	1.8
7/21/17	0:43	4.8	2.4	2.0
	2:09	4.6	2.3	2.0
7/22/17	0:54	4.9	3.6	1.4
	2:37	5.4	2.3	2.3
	5:15	6.7	1.4	4.8
	22:08	4.3	3.1	1.4
7/23/17	2:38	5.1	2.7	1.9
7/24/17	0:29	4.8	2	2.4
7/25/17	0:02	4.5	3.2	1.4
7/26/17	1:40	4.7	3.1	1.5
	22:24	4.5	3.4	1.3
7/27/17	12:12	4.5	4.4	1.0
7/28/17	2:20	4.9	3.5	1.4
	20:04	4.8	3.4	1.4
	23:22	5.5	2.9	1.9
7/29/17	0:26	4.6	3.2	1.4
	2:35	4.9	2.6	1.9
	19:14	5.2	3.3	1.6
	23:38	5.7	2.4	2.4
7/30/17	0:41	4.8	3.2	1.5
7/31/17	0:28	4.7	3.7	1.3
	2:23	7.1	2.1	3.4
	12:23	5.5	1.4	3.9
	19:19	5.2	2.9	1.8
	23:19	5.3	1.8	2.9
8/1/17	0:32	5	1.7	2.9
	1:08	4.9	2	2.5
	23:25	5.3	3.4	1.6
8/2/17	0:30	4.8	2.8	1.7
	20:57	3.9	4.4	0.9

	22:52	4.9	4.1	1.2
8/3/17	20:19	4.8	1.8	2.7
	23:30	5.2	2.2	2.4
8/4/17	23:04	4.4	2.8	1.6
8/5/17	1:30	3.9	2.8	1.4
8/10/17	19:28	4.4	4	1.1
8/11/17	0:14	5.3	2.5	2.1
	1:02	5.1	2.4	2.1
	14:21	4.4	3.4	1.3
	1837	4.9	3.2	1.5
	22:39	5.2	1.9	2.7
	23:45	5.1	2.7	1.9
8/12/17	20:44	4.3	3.6	1.2
	22:11	5.6	2.3	2.4
8/13/17	0:16	5.6	2.4	2.3
	19:45	4	4.2	1.0
	23:46	5	2.2	2.3
8/14/17	22:26	5.1	3.5	1.5
8/18/17	22:13	4.8	3.3	1.5
8/19/17	19:55	5.2	1.7	3.1
8/20/17	19:18	4.8	3.2	1.5
8/21/17	22:44	5.8	2.1	2.8
8/22/17	20:07	5.1	2.9	1.8
8/23/17	14:53	4.8	1.1	4.4
	19:07	3.9	3.4	1.1
	22:02	3.9	4.1	1.0
	23:19	4.1	3.2	1.3
8/24/17	1:29	5	1.1	4.5
	14:36	4.8	1.4	3.4
	15:48	4.7	2.8	1.7
	18:49	4.1	3.7	1.1
	22:57	4.4	1.4	3.1
8/25/17	0:37	4.2	2.2	1.9

	15:19	4.7	0.8	5.9
	16:42	4.7	2.1	2.2
	22:51	5	1	5.0
	23:58	4.4	1.8	2.4
8/26/17	1:53	4.5	1.8	2.5
	20:48	4.2	1.9	2.2
8/27/17	0:09	4.7	1.6	2.9
	12:15	4.3	2.2	2.0
	21:05	5.3	2	2.7
	21:47	4.3	1.9	2.3
8/28/17	0:05	4.6	2.2	2.1
	20:19	4.2	2.7	1.6
8/29/17	0:04	4.5	1.9	2.4
	23:31	4.5	1.2	3.8
8/30/17	1:35	4.2	1.9	2.2
	2:35	4.3	1.9	2.3
8/31/17	0:40	4.3	2.4	1.8
	20:31	4.4	1.6	2.8
9/1/17	0:03	4.6	1.6	2.9
	23:03	4.7	2.1	2.2
9/2/17	23:30	3.9	2.7	1.4
9/4/17	0:12	4.9	1.6	3.1
	23:31	4.3	3.2	1.3
9/5/17	2:31	4.2	2.6	1.6
9/6/17	0:13	4.6	1.6	2.9
9/7/17	0:21	4.3	1.3	3.3
	19:55	3.9	3.5	1.1
9/8/17	0:09	4.6	1.7	2.7
9/9/17	23:29	4.6	3.2	1.4
9/12/17	0:36	4.3	3.2	1.3
9/13/17	1:01	4.7	1.8	2.6
9/14/17	0:47	4.5	2.2	2.0
	23:47	4.5	2.3	2.0

9/15/17	23:07	4.4	3.3	1.3
9/16/17	23:36	4.4	3.4	1.3
9/17/17	23:11	4.7	2.7	1.7
9/18/17	1:19	4.4	2.4	1.8
9/19/17	0:23	4.8	1.5	3.2
	2:17	4.3	2.8	1.5
	19:45	4.6	2.5	1.8
9/20/17	0:23	4.4	2.8	1.6
9/21/17	0:58	4.3	2.5	1.7
	23:45	4.5	2.6	1.7
9/22/17	1:10	6.3	1.9	3.3
9/24/17	1:18	4.2	2	2.1
9/26/17	23:29	4.2	2.3	1.8
9/27/17	23:18	4.2	2.9	1.4
9/28/17	23:47	4.4	1.6	2.8
9/29/17	22:41	4.3	2.2	2.0
9/30/17	23:40	4.6	1.1	4.2
10/2/17	0:47	4.4	1.3	3.4
	23:51	4.4	2.7	1.6
10/4/17	23:40	4.8	1.6	3.0
10/6/17	22:53	4.3	2.3	1.9
10/7/17	23:36	3.9	3.5	1.1
10/8/17	19:56	4.2	3.6	1.2
10/9/17	12:26	5.1	0.8	6.4
10/10/17	19:46	3.9	2.5	1.6
10/11/17	18:51	3.7	4.3	0.9
	23:03	4.7	3.7	1.3
10/12/17	19:23	3.7	4.7	0.8
	23:54	4.2	2.3	1.8
10/13/17	22:21	4.3	1.8	2.4
10/14/17	22:43	4.3	3.7	1.2
10/15/17	23:25	4.8	2.7	1.8
10/19/17	18:46	3.7	2.3	1.6

10/20/17	19:26	3.6	3.8	0.9
10/22/17	19:20	3.6	2.4	1.5
10/25/17	19:14	4.1	1.9	2.2
10/28/17	22:42	3.9	3.1	1.3
10/29/17	19:49	4.4	2.1	2.1
	23:40	3.9	2.3	1.7
11/2/17	23:19	3.8	3.6	1.1
11/16/17	23:17	4.4	2	2.2
12/5/17	21:27	4.4	4.4	1.0

Table 1E, The Patient's Glucose, Ketone, and GKI Values for 2018

Date	Reading Time	Blood Ketone	Blood Ketone	GKI
1/21/18	15:49	4.2	2.6	1.6
	19:32	3.6	3.8	0.9
1/30/18	23:15	3.8	4.7	0.8
2/12/18	22:00	4.7	0.9	5.2
	11:52	4.1	3.2	1.3
2/23/18	23:19	4.1	3.2	1.3
		4.1	4.8	0.9
2/24/18	22:14	3.9	5.2	0.8
2/25/18	21:15	3.8	4.7	0.8
3/5/18	18:47	3.6	3.3	1.1
4/11/18	11:39	4.9	1.2	4.1
4/24/18	18:59	3.9	4.2	0.9
	19:40	3.7	4.3	0.9
	19:46	3.8	3.9	1.0
5/30/18	20:59	3.9	6.4	0.6
	21:02	3.8	5.7	0.7
6/20/18	19:29	4.1	2.9	1.4
7/10/18	19:41	4.7	0.9	5.2
7/11/18	0:11	4.2	2.2	1.9
	11:40	4.9	0.9	5.4

	14:11	4.9	1.3	3.8
	17:55	5.1	1	5.1
	18:35	4.9	1.3	3.8
	23:26	4.1	2.3	1.8
7/12/18	13:00	4.7	0.9	5.2
	14:35	4.2	1.1	3.8
	17:31	4.2	2.9	1.4
	18:38	4.2	0.5	8.4
	22:27	3.9	2.8	1.4
7/13/18	10:36	4.9	1.2	4.1
	12:25	5	1.6	3.1
	15:57	4.2	2.1	2.0
	20:07	4.3	2.2	2.0
	23:45	3.4	4.4	0.8
7/14/18	11:41	4.8	0.8	6.0
	15:58	4.2	2.1	2.0
	19:00	3.9	2.8	1.4
	20:26	4.1	2.9	1.4
7/15/18	9:43	4.3	1.4	3.1
	16:00	4.1	2.7	1.5
	20:14	3.9	4.5	0.9
7/16/18	10:28	5.1	1.4	3.6
	15:16	4.1	3.2	1.3
	23:09	3.8	2.3	1.7
7/17/18	10:46	4.8	1	4.8
	12:44	4.8	1.4	3.4
	17:24	3.7	3.2	1.2
	23:39	3.5	3.5	1.0
7/18/18	11:24	4.6	1.2	3.8
	14:57	4.2	2.4	1.8
	19:45	3.4	4.9	0.7
7/19/18	0:18	3.6	4.6	0.8
	9:10	4.6	2.6	1.8

	18:20	3.3	4.3	0.8
	23:35	3.7	2.8	1.3
7/20/18	9:31	4.4	1.7	2.6
	15:02	3.8	2.9	1.3
	20:46	3.7	3.8	1.0
7/21/18	23:57	5	1.8	2.8
	12:39	5	0.9	5.6
	16:02	4.1	2.8	1.5
	23:12	4.5	3.4	1.3
7/22/18	10:02	5.1	0.8	6.4
	23:35	4.2	2.1	2.0
7/24/18	23:17	3.9	1.8	2.2
7/25/18	11:51	4.9	0.8	6.1
	22:13	3.8	4.7	0.8
7/26/18	10:26	5	0.8	6.3
	22:16	4	3.8	1.1
7/29/18	18:10	3.8	5.5	0.7
7/31/18	22:22	4.2	3.1	1.4
8/2/18	18:18	4.3	3	1.4
9/16/18	17:09	4.2	4.1	1.0
9/17/18	18:39	4.1	3.3	1.2
9/20/18	15:06	5.1	1.1	4.6
	16:31	5	2.8	1.8
9/30/18	17:18	4.8	1.6	3.0
10/18/18	16:11	4.8	1.6	3.0
10/22/18	16:44	4.6	1.5	3.1
10/30/18	18:33	4.8	2.9	1.7
11/5/18	18:47	4.7	2.2	2.1
11/12/18	13:46	4.3	2.2	2.0
	16:17	3.8	1.8	2.1
11/13/18	15:34	3.9	2.8	1.4
11/14/18	8:57	4.3	2.2	2.0
11/15/18	8:13	4.2	2.9	1.4

11/16/18	8:30	4.6	2.3	2.0
	15:41	3.9	3.1	1.3
11/17/18	8:14	4.5	2.6	1.7
	17:34	4.1	3.8	1.1
11/18/18	8:55	4.7	2.2	2.1
	18:14	4.1	3.4	1.2
11/19/18	9:43	4.8	1.9	2.5
11/20/18	8:10	4.3	3	1.4
	18:06	3.9	3.7	1.1
11/21/18	8:09	4.1	3.1	1.3
11/22/18	8:13	4.3	2.5	1.7
11/23/18	8:30	4.5	2.1	2.1
	15:40	4.6	3.5	1.3
11/24/18	7:15	4.7	3	1.6
11/25/18	6:12	4.8	2.4	2.0
11/26/18	8:14	5.1	2.8	1.8
	20:21	3.6	4.9	0.7
11/27/18	8:11	5.3	3.2	1.7
	18:02	3.4	5.3	0.6
11/28/18	7:53	4.2	4.4	1.0
11/27/18	8:12	4.6	3.3	1.4
11/30/18	8:16	4.8	3.6	1.3
12/1/18	8:35	4.3	4.5	1.0
12/2/18	8:16	4.2	3.8	1.1
12/3/18	7:52	4	4.8	0.8
12/4/18	7:58	3.9	5.7	0.7
12/5/18	7:47	4.3	4.7	0.9
12/6/18	7:45	4.4	5.1	0.9
12/7/18	7:27	4.7	4.6	1.0
12/8/18	6:42	4.6	4.9	0.9
12/9/18	7:38	5.2	4.6	1.1
12/10/18	7:10	4.9	4.2	1.2
12/11/18	6:55	4.1	4.1	1.0

12/12/18	6:58	3.9	6	0.7
12/13/18	6:45	4.2	5.5	0.8
	16:12	3.7	6.4	0.6
12/14/18	6:26	4.3	4.5	1.0
12/15/18	6:30	4.2	5.2	0.8
12/16/18	6:30	3.9	5.3	0.7
12/17/18	6:04	4.1	5.8	0.7
12/18/18	6:03	3.8	5.5	0.7
12/25/18	5:20	4.1	4.8	0.9
12/26/18	8:12	4.6	3.9	1.2
12/27/18	5:22	4.1	4.8	0.9
12/28/18	5:47	4.3	4.9	0.9
12/29/18	6:00	4.2	4.1	1.0
12/30/18	6:03	4.3	5.1	0.8
12/31/18	6:03	4.2	4.9	0.9

Table 1F, The patient's Glucose, Ketone, and GKI Values for 2019

Date	Reading Time	Blood Glucose	Blood Ketone	GKI
01/01/19	7:04	4.5	5.2	0.9
01/02/19	5:57	4.3	4.6	0.9
01/03/19	6:03	3.9	4.9	0.8
01/04/19	5:57	4.3	3.7	1.2
01/05/19	5:47	3.8	5.1	0.7
01/06/19	5:51	4.2	4.6	0.9
01/07/19	5:53	4.4	5.3	0.8
01/08/19	5:50	3.8	5.3	0.7
01/09/19	5:51	4.1	4.4	0.9
01/10/19	8:11	4.1	3.7	1.1
01/11/19	8:07	4.1	3.6	1.1
01/12/19	7:41	4.2	4.7	0.9
01/13/19	7:00	4.4	4.6	1.0
01/14/19	7:25	4.3	3.6	1.2

01/15/19	6:31	4.4	3.9	1.1
01/16/19	6:08	4.3	4.4	1.0
01/18/19	8:22	4.6	4.2	1.1
01/20/19	6:18	3.7	4.9	0.8
01/21/19	6:20	4	4.4	0.9
01/22/19	8:31	4.4	4.8	0.9
01/23/19	6:19	3.8	4.9	0.8
01/24/19	6:11	4.3	4.3	1.0
01/25/19	8:17	4.4	4.3	1.0
01/26/19	5:48	4.6	3.2	1.4
01/27/19	6:21	4.6	3.7	1.2
01/28/19	6:22	4.6	3.4	1.4
02/11/19	6:11	3.9	4.6	0.8
02/13/19	6:09	3.6	4.7	0.8
02/20/19	8:38	4.7	3.6	1.3
02/28/19	8:25	4.6	3.1	1.5
03/01/19	8:19	4.6	2.8	1.6
03/04/19	7:49	4.9	1.4	3.5
03/06/19	8:30	4.7	2.3	2.0
03/08/19	8:34	4.6	2.6	1.8
03/11/19	8:24	4.7	2.1	2.2
03/12/19	8:18	4.6	1.9	2.4
	17:35	3.6	4.7	0.8
03/15/19	8:30	4.9	1	4.9
		4.2	3.1	1.4
03/16/19	8:16	4.6	0.9	5.1
	11:19	4.8	1	4.8
	16:16	3.1	3.5	0.9
03/17/19	8:15	4.3	1.1	3.9
03/18/19	8:13	4.8	1.1	4.4
03/19/19	8:15	4.1	2.1	2.0
03/20/19	8:01	4.4	2.3	1.9
03/21/19	7:25	4.5	1.9	2.4

03/22/19	8:31	4.7	1.9	2.5
03/25/19	8:25	5.2	0.8	6.5
	20:52	5.1	1.5	3.4
03/26/19	6:51	4.1	1.6	2.6
03/27/19	6:51	4.8	1.7	2.8
03/28/19	8:13	4.2	2	2.1
03/29/19	9:42	3.8	1.8	2.1
03/30/19	8:19	4.1	2.7	1.5
04/01/19	8:37	4.8	2.9	1.7
04/02/19	9:20	4.1	3.3	1.2
04/03/19	8:16	4.4	3.7	1.2
04/04/19	8:21	4.2	3.2	1.3
04/05/19	8:54	4.7	2.9	1.6
	16:16	3.2	5.2	0.6
04/06/19	9:28	5.1	1.2	4.3
	17:50	4.1	3.8	1.1
04/08/19	8:27	4.7	1.5	3.1
	14:10	4.3	2.7	1.6
04/09/19	8:36	4.9	2.3	2.1
	16:38	4.4	2.4	1.8
04/10/19	8:33	4.7	1.3	3.6
	13:27	4.4	3.4	1.3
04/11/19	6:48	5.2	1.2	4.3
04/12/19	7:26	4.6	1.2	3.8
04/14/19	9:09	4.7	2.3	2.0
04/18/19	8:46	5	2.2	2.3
05/08/19	8:59	5	2	2.5
05/09/19	7:56	4.3	3.4	1.3
05/10/19	8:38	5.1	1.3	3.9
05/11/19	18:56	5.9	2.3	2.6
		4.9	2.1	2.3
05/12/19	10:24	4.5	2.3	2.0
05/13/19	8:13	4.6	2.8	1.6

05/14/19	8:27	4.4	2.9	1.5
05/20/19	9:39	5.1	2.8	1.8
	20:02	4.3	1.8	2.4
06/17/19	9:00	5.4	1	5.4
06/21/19	10:08	5.2	0.6	8.7
07/17/19	8:37	4.9	1.4	3.5
08/27/19	9:26	4.1	2.9	1.4
08/28/19	8:33	4.2	2.9	1.4
08/29/19	8:22	4.7	2.3	2.0
08/30/19	8:00	4.2	2.8	1.5
09/03/19	20:24	3.7	4.1	0.9
09/10/19	9:35	4.7	1.1	4.3
09/24/19	8:35	4.4	2.1	2.1
09/25/19	8:26	4.5	1.8	2.5
